# A Web-Based Calculator to Predict Early Death Among Patients With Bone Metastasis Using Machine Learning Techniques: Development and Validation Study

**DOI:** 10.2196/47590

**Published:** 2023-10-23

**Authors:** Mingxing Lei, Bing Wu, Zhicheng Zhang, Yong Qin, Xuyong Cao, Yuncen Cao, Baoge Liu, Xiuyun Su, Yaosheng Liu

**Affiliations:** 1 Senior Department of Orthopedics, The Fourth Medical Center of PLA General Hospital Beijing China; 2 Department of Orthopedics, Hainan Hospital of Chinese PLA General Hospital Hainan China; 3 Chinese PLA Medical School Beijing China; 4 Department of Orthopedics, The First Medical Center of PLA General Hospital Beijing China; 5 Department of Joint and Sports Medicine Surgery, The Second Affiliated Hospital of Harbin Medical University Harbin China; 6 Department of Orthopedics, Beijing Tiantan Hospital, Capital Medical University Beijing China; 7 Intelligent Medical Innovation institute, Southern University of Science and Technology Hospital Shenzhen China; 8 Department of Orthopedics, The Fifth Medical Center of PLA General Hospital Beijing China; 9 National Clinical Research Center for Orthopedics, Sports Medicine & Rehabilitation PLA General Hospital Beijing China

**Keywords:** bone metastasis, early death, machine learning, prediction model, local interpretable model–agnostic explanation

## Abstract

**Background:**

Patients with bone metastasis often experience a significantly limited survival time, and a life expectancy of <3 months is generally regarded as a contraindication for extensive invasive surgeries. In this context, the accurate prediction of survival becomes very important since it serves as a crucial guide in making clinical decisions.

**Objective:**

This study aimed to develop a machine learning–based web calculator that can provide an accurate assessment of the likelihood of early death among patients with bone metastasis.

**Methods:**

This study analyzed a large cohort of 118,227 patients diagnosed with bone metastasis between 2010 and 2019 using the data obtained from a national cancer database. The entire cohort of patients was randomly split 9:1 into a training group (n=106,492) and a validation group (n=11,735). Six approaches—logistic regression, extreme gradient boosting machine, decision tree, random forest, neural network, and gradient boosting machine—were implemented in this study. The performance of these approaches was evaluated using 11 measures, and each approach was ranked based on its performance in each measure. Patients (n=332) from a teaching hospital were used as the external validation group, and external validation was performed using the optimal model.

**Results:**

In the entire cohort, a substantial proportion of patients (43,305/118,227, 36.63%) experienced early death. Among the different approaches evaluated, the gradient boosting machine exhibited the highest score of prediction performance (54 points), followed by the neural network (52 points) and extreme gradient boosting machine (50 points). The gradient boosting machine demonstrated a favorable discrimination ability, with an area under the curve of 0.858 (95% CI 0.851-0.865). In addition, the calibration slope was 1.02, and the intercept-in-large value was −0.02, indicating good calibration of the model. Patients were divided into 2 risk groups using a threshold of 37% based on the gradient boosting machine. Patients in the high-risk group (3105/4315, 71.96%) were found to be 4.5 times more likely to experience early death compared with those in the low-risk group (1159/7420, 15.62%). External validation of the model demonstrated a high area under the curve of 0.847 (95% CI 0.798-0.895), indicating its robust performance. The model developed by the gradient boosting machine has been deployed on the internet as a calculator.

**Conclusions:**

This study develops a machine learning–based calculator to assess the probability of early death among patients with bone metastasis. The calculator has the potential to guide clinical decision-making and improve the care of patients with bone metastasis by identifying those at a higher risk of early death.

## Introduction

Accurately estimating the survival outcome of patients with bone metastasis is crucial for guiding appropriate therapeutic interventions [1-3]. Current therapeutic strategies for bone metastasis primarily involve radiation, chemotherapy, and surgery, often in combination [1]. However, it is generally advised that patients with a life expectancy of <3 months should not undergo extensive invasive surgeries, as the potential risks may outweigh the benefits [3]. Conversely, patients with a relatively longer life expectancy may benefit from surgical interventions for bone fracture or spinal instability, rather than relying solely on radiotherapy or best supportive care [4]. It is worth noting that the duration of radiotherapy in patients with bone metastasis is also dependent on survival prediction. Patients with a more favorable life expectancy may undergo a longer course of radiotherapy because shorter courses have been associated with higher rates of in-field recurrence [5].

In recent years, advancements in therapeutic modalities have led to an increasing number of surgical interventions for bone metastasis [6,7], such as osteosynthesis for extremity bone metastasis and excisional surgery for spinal metastasis. These procedures aim to maintain or improve the functional outcomes of patients and enhance their quality of life. However, striking a balance between the benefits and potential harms of surgery remains a challenge. Survival prediction plays a crucial role in guiding surgical strategies [3]. For patients with a relatively short life expectancy, simpler fixation techniques for extremity bone metastasis or posterior decompression and fixation for spinal metastasis are recommended because these approaches are associated with fewer complications but an increased risk of implant failure if patients have a better survival prognosis. In contrast, excisional surgery for spinal metastasis and prosthetic replacement for bone metastasis in the extremities are preferred for patients with a longer life expectancy [1,3].

Previous studies on prognostic factors for patients with bone metastasis have been limited by bias in terms of patient’s selection [8], data sample, and modeling methodology, which impacted their accuracy and generalization. However, the emergence of machine learning techniques has provided new opportunities to improve prognostic models in various aspects of cancer, including early diagnosis, treatment, and understanding biological processes [9-11]. Machine learning involves the application of algorithms that can explore nonlinear associations between variables and outcomes, allowing for the calculation of risk probabilities in different data sets. Machine learning–based models have demonstrated superior accuracy compared with standard eligibility criteria [11] and other nonmachine learning strategies [12,13].

Therefore, this study aimed to develop and validate a more accurate machine learning–based prediction model to assess the risk of early death among patients with bone metastasis. This study hypothesized that by identifying risk factors significantly associated with early death and using machine learning algorithms, an optimal prediction model could be developed. This model would enhance the accuracy and generalizability of prognostic assessments for patients with bone metastasis, ultimately improving clinical decision-making and patient outcomes.

## Methods

### Inclusion and Exclusion Criteria

This study analyzed 186,069 patients with bone metastasis between 2010 and 2019 from the Surveillance, Epidemiology, and End Results (SEER) database. The SEER database is a reliable and authoritative source of cancer statistics, covering approximately 28% of the population in the United States. It is supported by the Surveillance Research Program in the National Cancer Institute and encompasses data from various locations and sources across the country.

In this study, patients with bone metastasis were extracted from the database (2010-2019) using the SEER*Stat software (version 8.4.0.1; National Institutes of Health National Cancer Institute). The inclusion criteria were patients with bone metastasis, whereas the exclusion criteria were applied to remove patients who were aged ≤18 years, had unknown survival time, died owing to causes other than cancer, had unknown or missing cause of death, had missing data, or were alive with a follow-up time of only ≤3 months. We excluded patients with a follow-up period of <3 months from the study, and this decision was made because the primary outcome of the study was early death, and we could not ascertain whether a patient would die within 3 months after their diagnosis of bone metastases based on such a short follow-up period.

**Figure 1:**
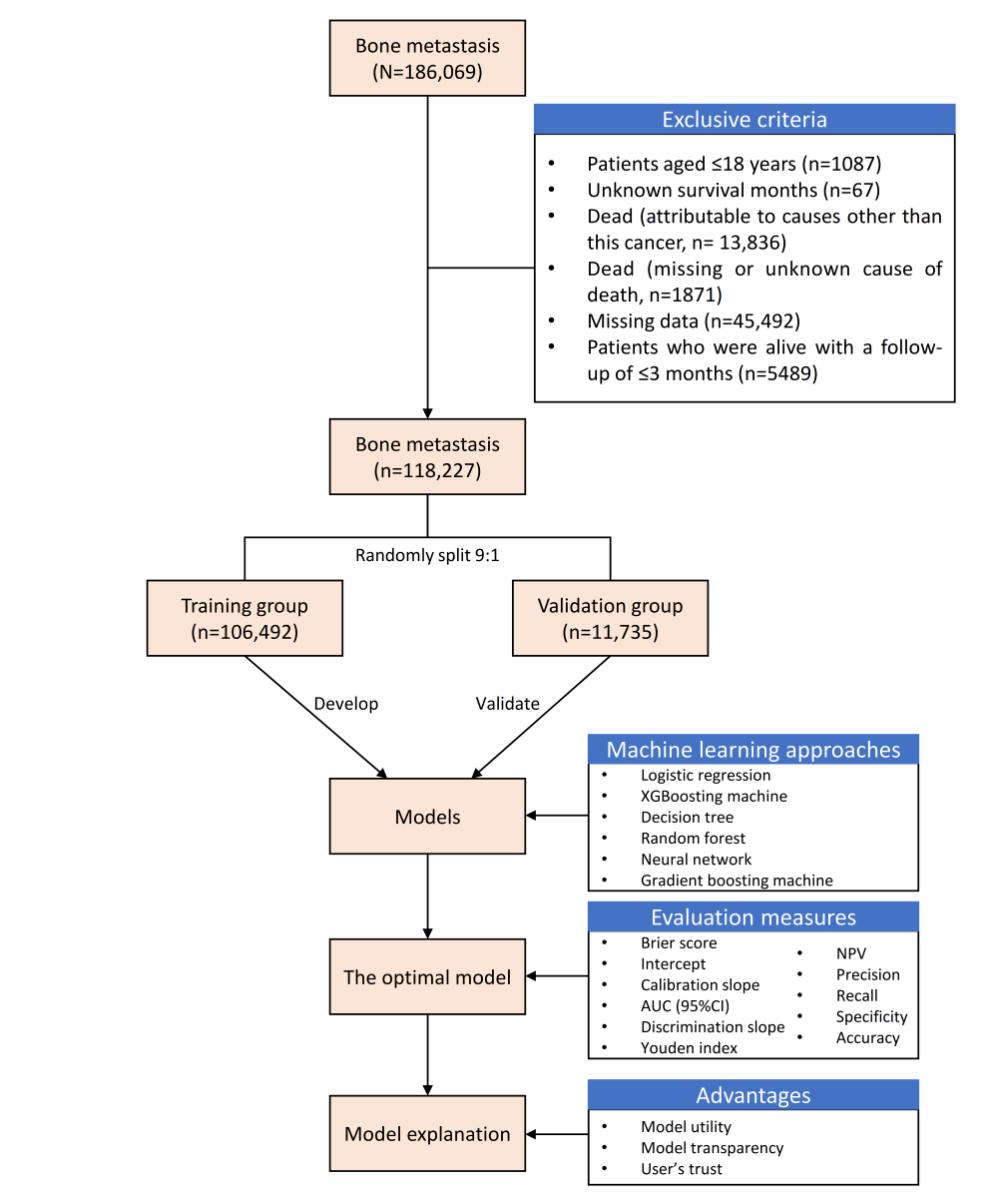
Patient flowchart and study design. AUC: area under curve; NPV: negative predictive value.

Figure 1 illustrates the flowchart of patients and the study design used in this research, which provided a visual representation of how patients were selected and included in the study as well as the overall design and methodology used. This study included 118,227 patients with bone metastasis based on the inclusion and exclusion criteria. We included a diverse range of patients with bone metastasis, encompassing both spinal and extremity cases, and we also considered various treatment modalities including surgery, radiotherapy, chemotherapy, and no therapy. This comprehensive inclusion of patients with different characteristics and treatment histories enables the prediction model to be applicable to a broader population rather than being limited to a specific subset of patients.

The patients were randomly split 9:1 into a training group (n=106,492) and a validation group (n=11,735). Patients in the training group were used to train and optimize the prediction models, and patients in the validation group were used to assess and validate the prediction performance of the models. According to the inclusion and exclusion criteria, patients (n=332) from the Fifth Medical Center of Chinese PLA General Hospital were included in the external validation group, and external validation was also performed in this set.

### Ethical Considerations

This study was approved by the Ethics Committee of the Fifth Medical Center of Chinese PLA General Hospital (KY-2019-12-59). Informed written consent was obtained from all patients, and strict measures were implemented to ensure the anonymity and confidentiality of the data during the analysis process. The study adhered to the principles outlined in the Declaration of Helsinki, which provides ethical guidelines for medical research involving human participants. Our findings were reported in compliance with the Standards for Developing and Reporting Machine Learning Predictive Models in Biomedical Research [14].

### Potential Risk Variables

A total of 14 potential risk variables were included in the analysis, including patient’s demographics, tumor conditions, metastatic conditions, and clinical therapeutic interventions. The demographics included age, sex (female vs male), race (Black vs White vs others vs unknown), marital status (divorced vs married [including common law] vs separated vs single [never married] vs widowed vs unmarried or domestic partner vs unknown), and rural-urban continuum (metropolitan counties vs nonmetropolitan counties vs unknown); tumor conditions included primary site (slow vs moderate vs rapid growth), tumor stage (T stage; T0 vs T1 vs T2 vs T3 vs T4 vs TX [unknown T stage]), and node stage (N stage; N0 vs N1 vs N2 vs N3 vs NX [unknown N stage]); and metastatic conditions included brain metastasis (no vs unknown vs yes), liver metastasis (no vs unknown vs yes), and lung metastasis (no vs unknown vs yes). The clinical therapeutic interventions included cancer-directed surgery (no vs unknown vs yes), radiation (none or unknown vs yes), and chemotherapy (none or unknown vs yes).

All patients entered our database after their diagnosis of bone metastases owing to cancer. Early death was defined as patients who died at or within 3 months [15,16] after the diagnosis of bone metastases, and the death was attributed to causes related to their cancer. T and N stages were recorded based on the combination of the American Joint Committee on Cancer and Extent of Disease classification. The primary site of cancer was divided according to a previous study [8]. Table S1 in Multimedia Appendix 1 provides the detailed information regarding the classification of primary cancers.

### Model Development and Validation

This study used multiple logistic regression analysis of clinical characteristics for predicting early death, supplemented by subgroup analysis of clinical characteristics stratified by early death, to conduct variable selection. Significant clinical characteristics in the multivariate analysis served as input features to train and optimize the models. As for machine learning approaches, in the training group, 6 algorithms including logistic regression, extreme gradient boosting (XGBoosting) machine, decision tree, random forest, neural network, and gradient boosting machine were used to train and optimize the models [17-21]. The introduction of the 6 machine learning algorithms is summarized in Table S2 in Multimedia Appendix 1. To ensure robustness and reproducibility, all the models were provided with identical input features. To optimize the performance of each model, an extensive exploration of the hyperparameter space was conducted through both grid and random searches. The area under the curve (AUC), a widely accepted metric for classification tasks, was used as the objective function for hyperparameter optimization. To strike a balance between model complexity and generalization ability, careful consideration was given to prevent both overfitting and underfitting. As such, the search space for the hyperparameters was deliberately defined with generous upper and lower bounds. For instance, the decision tree depth was constrained within the range of 2 to 100, accounting for a wide spectrum of potential tree structures. Optimal model parameters were obtained after a grid search or random hyperparameter search using 5-fold cross-validation after 100 iterations of bootstraps.

The prediction performance of the models was assessed using 11 measures in the validation group: Brier score, intercept-in-large, calibration slope, AUC, discrimination slope, specificity, negative predictive value, precision, recall, Youden index, and accuracy. In addition, the clinical usefulness of the models was evaluated using decision curve analysis after calculating the net benefits in a range of threshold probability. The metrics were scored by sorting them based on their prediction performance, as per the findings of a previous study [16]. On the basis of each evaluation metric, the highest score was assigned to the best-performing model, followed by the next best model. The overall score of the model was the sum of the scores of each metric. The total score of the scale was 66 points, with a score of >52 indicating excellent prediction performance. The heat map was conducted to show the data using pheatmap package in R language software (R Foundation for Statistical Computing). External validation was performed using the optimal machine learning model.

### Model Explainability and Variable Importance

The Local Interpretable Model-Agnostic Explanations (LIME) technique was used to enhance the explainability of the best model, thereby promoting its clinical utility and transparency [22]. LIME allowed the calculation of the risk probability of early death and facilitated a deeper understanding of how the predicted probability changes with different observations by assigning individual weights to each variable. This approach significantly increased users’ trust in the prediction model through reasonable model explanations and enhanced model transparency.

In addition, the importance of predictors was assessed using the SHAP (Shapley Additive Explanations) method [19]. This evaluation enabled the ranking of variables based on their contributions to the outcome, providing insights into which factors had the most significant impact on the prediction. By using SHAP, a comprehensive understanding of the relative importance of different variables in determining the risk of early death among patients with bone metastasis was obtained.

### Model Risk Stratification

To achieve risk stratification, this study used the average threshold derived from the 6 approaches as the optimal threshold. On the basis of this threshold value, patients were classified into 2 distinct groups: a low-risk group and a high-risk group, as defined by each approach. Specifically, patients with a predicted probability equal to or less than the threshold were assigned to the low-risk group, whereas patients with a predicted probability greater than the threshold were assigned to the high-risk group. This approach allowed for the differentiation of patients based on their predicted risk of early death, facilitating targeted interventions and personalized management strategies.

### Development of the Web-Based Calculator

Using the algorithm of the optimal model from this study, we developed an web-based calculator to predict the probability of early death in patients with bone metastasis. This calculator is accessible through the *Streamlit* application and has been designed with 4 main sections. The first section of the calculator allows users to input the relevant parameters and select the model variables. This interactive panel enables users to customize the prediction based on specific patient characteristics and treatment options. The second section of the calculator displays the predicted probability of early mortality. Once the user inputs the necessary parameters, the calculator generates an estimate of the likelihood of early death for the given patient. This information can provide valuable insights into clinical decision-making. The third section of the calculator provides detailed information about the model itself. This section offers an overview of the algorithm used, the data set used, and the validation process. It aims to enhance transparency and provide users with a clear understanding of how the predictions are generated. The fourth section of the calculator offers recommendations for therapeutic strategies based on the predicted risk of early death. This section provides guidance on potential treatment options, taking into account the patient’s risk level and the goal of palliative pain relief.

### Statistical Analysis

In this study, quantitative data were summarized as mean and SD, and qualitative data were presented as proportions. The difference comparison was performed using 2-tialed *t* test for the quantitative variables and using adjusted continuity chi-square test for the qualitative variables. Multivariate logistic regression analysis was conducted to identify the significant variables associated with early death. Kaplan-Meier survival curves were plotted according to risk stratification among all approaches using “survival” and “survminer” package. Survival difference between the low-risk and high-risk groups were compared using the log-rank test. The association between age and early death was investigated using an automatic machine learning method. This association was visualized according to the deciles of all patients. Machine learning and model explainability were conducted using Python (version 3.9.7; Python Software Foundation); visualization and statistical analysis were both performed using R programming language (version 4.1.2). All *P* values were 2-tailed, and a *P* value of <.05 was considered statistically significant.

## Results

### Patient’s Basic Demographics and Clinical Characteristics

A total of 186,069 patients were included in the analysis, with a mean age of 67.10 (SD 12.39) years. Most patients were male (68,296/118,227, 57.77%), White (94,061/118,227, 79.56%), and married (60,812/118,227, 51.44%). Rapid-growth primary cancer accounted for 62.49% (73,881/118,227) of patients, followed by slow growth (32,309/118,227, 27.33%) and moderate growth (12,037/118,227, 10.18%). A significant proportion of the patients were in the advanced stages, with 41.57% (49,167/118,227) in the T3 and T4 stages and 35.99% (42,553/118,227) in the N2 and N3 stages. In the entire cohort, early death occurred in 36.63% (43,305/118,227) of patients. Multimedia Appendix 2 illustrates the incidence of early death over the course of the study period, which revealed a consistent and stable pattern of occurrence per year. The therapeutic interventions and organ metastases are summarized in Table 1. It also showed the distribution of clinical characteristics between the training and validation groups, indicating that the 2 groups were comparable.

**Table 1:** Patient’s demographics and clinical characteristics among all patients with bone metastasis and a comparison between patients in the training and validation groups (N=118,227).

Characteristics		Overall	Training group (n=106,492)	Validation group (11,735)	*P* value	
Age, mean (SD)		67.10 (12.39)	67.09 (12.40)	67.23 (12.28)	.26	
**Sex, n (%)**						.62
	Female	49,931 (42.23)	45,001 (42.26)	4930 (42.01)		
	Male	68,296 (57.77)	61,491 (57.74)	6805 (57.99)		
**Race, n (%)**						.71
	Black	14,135 (11.96)	12,736 (11.96)	1399 (11.92)		
	White	94,061 (79.56)	84,740 (79.57)	9321 (79.43)		
	Others	9715 (8.22)	8727 (8.19)	988 (8.42)		
	Unknown	316 (0.27)	289 (0.27)	27 (0.23)		
**Marital status, n (%)**						.26
	Divorced	13,413 (11.35)	12,125 (11.39)	1288 (10.98)		
	Married (including common law)	60,812 (51.44)	54,734 (51.4)	6078 (51.79)		
	Separated	1479 (1.25)	1306 (1.23)	173 (1.47)		
	Single (never married)	20,419 (17.27)	18,406 (17.28)	2013 (17.15)		
	Widowed	15,946 (13.49)	14,372 (13.5)	1574 (13.41)		
	Unmarried or domestic partner	438 (0.37)	399 (0.37)	39 (0.33)		
	Unknown	5720 (4.84)	5150 (4.84)	570 (4.86)		
**Rural-urban continuum, n (%)**						.08
	Metropolitan counties	101,904 (86.19)	91,864 (86.26)	10,040 (85.56)		
	Nonmetropolitan counties	16,175 (13.68)	14,498 (13.61)	1677 (14.29)		
	Unknown	148 (0.13)	130 (0.12)	18 (0.15)		
**Primary site, n (%)**						.78
	Slow growth	32,309 (27.33)	29,115 (27.34)	3194 (27.22)		
	Moderate growth	12,037 (10.18)	10,860 (10.2)	1177 (10.03)		
	Rapid growth	73,881 (62.49)	66,517 (62.46)	7364 (62.75)		
**T stage^a^, n (%)**						.98
	T0	1730 (1.46)	1556 (1.46)	174 (1.48)		
	T1	15,293 (12.94)	13,782 (12.94)	1511 (12.88)		
	T2	25,004 (21.15)	22,489 (21.12)	2515 (21.43)		
	T3	20,998 (17.76)	18,912 (17.76)	2086 (17.78)		
	T4	28,169 (23.83)	25,384 (23.84)	2785 (23.73)		
	TX^b^	27,033 (22.87)	24,369 (22.88)	2664 (22.7)		
**N stage^c^, n (%)**						.92
	N0	35,017 (29.62)	31,527 (29.61)	3490 (29.74)		
	N1	24,271 (20.53)	21,901 (20.57)	2370 (20.2)		
	N2	27,961 (23.65)	25,168 (23.63)	2793 (23.8)		
	N3	14,592 (12.34)	13,136 (12.34)	1456 (12.41)		
	NX^d^	16,386 (13.86)	14,760 (13.86)	1626 (13.86)		
**Brain metastasis, n (%)**						.70
	No	98,208 (83.07)	88,492 (83.1)	9716 (82.8)		
	Unknown	3955 (3.35)	3553 (3.34)	402 (3.43)		
	Yes	16,064 (13.59)	14,447 (13.57)	1617 (13.78)		
**Liver metastasis, n (%)**						.12
	No	83,629 (70.74)	75,414 (70.82)	8215 (70)		
	Unknown	3151 (2.67)	2816 (2.64)	335 (2.85)		
	Yes	31,447 (26.6)	28,262 (26.54)	3185 (27.14)		
**Lung metastasis, n (%)**						.24
	No	83,055 (70.25)	74,813 (70.25)	8242 (70.23)		
	Unknown	4272 (3.61)	3817 (3.58)	455 (3.88)		
	Yes	30,900 (26.14)	27,862 (26.16)	3038 (25.89)		
**Cancer-directed surgery, n (%)**						.70
	No	106,096 (89.74)	95,548 (89.72)	10,548 (89.88)		
	Unknown	599 (0.51)	545 (0.51)	54 (0.46)		
	Yes	11,532 (9.75)	10,399 (9.77)	1133 (9.65)		
**Radiation, n (%)**						.80
	None or unknown	71,422 (60.41)	64,346 (60.42)	7076 (60.3)		
	Yes	46,805 (39.59)	42,146 (39.58)	4659 (39.7)		
**Chemotherapy, n (%)**						>.99
	None or unknown	63,740 (53.91)	57,413 (53.91)	6327 (53.92)		
	Yes	54,487 (46.09)	49,079 (46.09)	5408 (46.08)		
**Early death, n (%)**						.49
	No	74,922 (63.37)	67,451 (63.34)	7471 (63.66)		
	Yes	43,305 (36.63)	39,041 (36.66)	4264 (36.34)		

^a^T stage: tumor stage.

^b^TX: unknown tumor stage.

^c^N stage: node stage.

^d^NX: unknown node stage.

### Model Development

In the training group, a comparison of variables between patients with and without early death was performed (Table 2). According to multivariate analysis, age, sex, race, marital status, rural-urban continuum, primary site, T stage, N stage, brain metastasis, liver metastasis, lung metastasis, cancer-directed surgery, radiation, and chemotherapy were all significantly associated with early death (Table 3) and were included as input features for model training and optimization. The full parameter weights of the machine learning algorithms are summarized in Table S3 in Multimedia Appendix 1.

**Table 2:** A comparison of clinical characteristics between patients with and without early death among patients with bone metastasis (N=106,492).

Characteristics		Overall	Early death			*P* value	
			No (n=67,451)	Yes (n=39,041)			
Age, mean (SD)		67.09 (12.40)	65.58 (12.57)	69.70 (11.66)	<.001		
**Sex, n (%)**							<.001
	Female	45,001 (42.26)	28,941 (42.91)	16,060 (41.14)			
	Male	61,491 (57.74)	38,510 (57.09)	22,981 (58.86)			
**Race, n (%)**							<.001
	Black	12,736 (11.96)	8379 (12.42)	4357 (11.16)			
	White	84,740 (79.57)	52,797 (78.27)	31,943 (81.82)			
	Others	8727 (8.19)	6034 (8.95)	2693 (6.9)			
	Unknown	289 (0.27)	241 (0.36)	48 (0.12)			
**Marital status, n (%)**							<.001
	Divorced	12,125 (11.39)	7293 (10.81)	4832 (12.38)			
	Married (including common law)	54,734 (51.4)	36,645 (54.33)	18,089 (46.33)			
	Separated	1306 (1.23)	816 (1.21)	490 (1.26)			
	Single (never married)	18,406 (17.28)	11,479 (17.02)	6927 (17.74)			
	Widowed	14,372 (13.5)	7550 (11.19)	6822 (17.47)			
	Unmarried or domestic partner	399 (0.37)	274 (0.41)	125 (0.32)			
	Unknown	5150 (4.84)	3394 (5.03)	1756 (4.5)			
**Rural-urban continuum, n (%)**							<.001
	Metropolitan counties	91,864 (86.26)	58,832 (87.22)	33,032 (84.61)			
	Nonmetropolitan counties	14,498 (13.61)	8542 (12.66)	5956 (15.26)			
	Unknown	130 (0.12)	77 (0.11)	53 (0.14)			
**Primary site, n (%)**							<.001
	Slow growth	29,115 (27.34)	26,096 (38.69)	3019 (7.73)			
	Moderate growth	10,860 (10.2)	7034 (10.43)	3826 (9.8)			
	Rapid growth	66,517 (62.46)	34,321 (50.88)	32,196 (82.47)			
**T stage^a^, n (%)**							<.001
	T0	1556 (1.46)	963 (1.43)	593 (1.52)			
	T1	13,782 (12.94)	10,083 (14.95)	3699 (9.47)			
	T2	22,489 (21.12)	15,511 (23)	6978 (17.87)			
	T3	18,912 (17.76)	11,739 (17.4)	7173 (18.37)			
	T4	25,384 (23.84)	14,999 (22.24)	10,385 (26.6)			
	TX^b^	24,369 (22.88)	14,156 (20.99)	10,213 (26.16)			
**N stage^c^, n (%)**							<.001
	N0	31,527 (29.61)	21,543 (31.94)	9984 (25.57)			
	N1	21,901 (20.57)	15,873 (23.53)	6028 (15.44)			
	N2	25,168 (23.63)	13,470 (19.97)	11,698 (29.96)			
	N3	13,136 (12.34)	7879 (11.68)	5257 (13.47)			
	NX^d^	14,760 (13.86)	8686 (12.88)	6074 (15.56)			
**Brain metastasis, n (%)**							<.001
	No	88,492 (83.1)	58,123 (86.17)	30,369 (77.79)			
	Unknown	3553 (3.34)	1899 (2.82)	1654 (4.24)			
	Yes	14,447 (13.57)	7429 (11.01)	7018 (17.98)			
**Liver metastasis, n (%)**							<.001
	No	75,414 (70.82)	52,075 (77.2)	23,339 (59.78)			
	Unknown	2816 (2.64)	1621 (2.4)	1195 (3.06)			
	Yes	28,262 (26.54)	13,755 (20.39)	14,507 (37.16)			
**Lung metastasis, n (%)**							<.001
	No	74,813 (70.25)	50,271 (74.53)	24,542 (62.86)			
	Unknown	3817 (3.58)	2156 (3.2)	1661 (4.25)			
	Yes	27,862 (26.16)	15,024 (22.27)	12,838 (32.88)			
**Cancer-directed surgery, n (%)**							<.001
	No	95,548 (89.72)	58,079 (86.11)	37,469 (95.97)			
	Unknown	545 (0.51)	424 (0.63)	121 (0.31)			
	Yes	10,399 (9.77)	8948 (13.27)	1451 (3.72)			
**Radiation, n (%)**							<.001
	None or unknown	64,346 (60.42)	38,402 (56.93)	25,944 (66.45)			
	Yes	42,146 (39.58)	29,049 (43.07)	13,097 (33.55)			
**Chemotherapy, n (%)**							<.001
	None or unknown	57,413 (53.91)	27,795 (41.21)	29,618 (75.86)			
	Yes	49,079 (46.09)	39,656 (58.79)	9423 (24.14)			

^a^T stage: tumor stage.

^b^TX: unknown tumor stage.

^c^N stage: node stage.

^d^NX: unknown node stage.

**Table 3:** Multivariate analysis of characteristics for predicting early death among patients with bone metastasis.

Characteristics			Odds ratio (95% CI)		*P* value
Age			1.017 (1.015-1.018)		<.001
**Sex**					
	Female	Reference		—^a^	
	Male	1.123 (1.086-1.161)		<.001	
**Race**					
	Black	Reference		—	
	White	1.036 (0.985-1.089)		.17	
	Others	0.761 (0.707-0.819)		<.001	
	Unknown	0.372 (0.256-0.542)		<.001	
**Marital status**					
	Divorced	Reference		—	
	Married (including common law)	0.838 (0.797-0.882)		<.001	
	Separated	1.057 (0.912-1.224)		.46	
	Single (never married)	1.025 (0.965-1.088)		.42	
	Unknown	0.795 (0.729-0.866)		<.001	
	Unmarried or domestic partner	0.781 (0.597-1.022)		.07	
	Widowed	0.975 (0.915-1.040)		.44	
**Rural-urban continuum**					
	Metropolitan counties	Reference		—	
	Nonmetropolitan counties	1.188 (1.135-1.243)		<.001	
	Unknown	1.863 (1.184-2.931)		.007	
**Primary site**					
	Rapid growth	1.577 (1.492-1.667)		<.001	
	Moderate growth	Reference		—	
	Slow growth	0.105 (0.098-0.112)		<.001	
**T stage^b^**					
	T0	Reference		—	
	T1	0.937 (0.818-1.073)		.35	
	T2	1.119 (0.980-1.278)		.10	
	T3	1.189 (1.041-1.358)		.01	
	T4	1.373 (1.203-1.566)		<.001	
	TX^c^	1.398 (1.226-1.594)		<.001	
**N stage^d^**					
	N0	Reference		—	
	N1	1.133 (1.080-1.189)		<.001	
	N2	1.237 (1.181-1.295)		<.001	
	N3	1.143 (1.082-1.208)		<.001	
	NX^e^	1.181 (1.119-1.247)		<.001	
**Brain metastasis**					
	No	Reference		—	
	Unknown	1.023 (0.921-1.135)		.67	
	Yes	1.546 (1.476-1.619)		<.001	
**Liver metastasis**					
	No	Reference		—	
	Unknown	1.002 (0.891-1.1270)		.98	
	Yes	1.930 (1.862-2.002)		<.001	
**Lung metastasis**					
	No	Reference		—	
	Unknown	1.104 (1.001-1.218)		.047	
	Yes	1.390 (1.339-1.442)		<.001	
**Cancer-directed surgery**					
	No	Reference		—	
	Unknown	0.546 (0.422-0.707)		<.001	
	Yes	0.347 (0.324-0.372)		<.001	
**Radiation**					
	None or unknown	Reference		—	
	Yes	0.658 (0.636-0.681)		<.001	
**Chemotherapy**					
	None or unknown	Reference		—	
	Yes	0.109 (0.105-0.113)		<.001	

^a^Not applicable.

^b^T stage: tumor stage.

^c^TX: unknown tumor stage.

^d^N stage: node stage.

^e^NX: unknown node stage.

### Model Validation

The AUC of the gradient boosting machine and XGBoosting machine was 0.858 (95% CI 0.851-0.865; Multimedia Appendix 3), followed by random forest and neural network (both 0.856, 95% CI 0.849-0.863). The calibration slopes of the logistic regression, XGBoosting machine, decision tree, random forest, neural network, and gradient boosting machine were 1.05, 1.03, 0.97, 1.13, 0.97, and 1.02, respectively (Multimedia Appendix 4).

**Figure 2:**
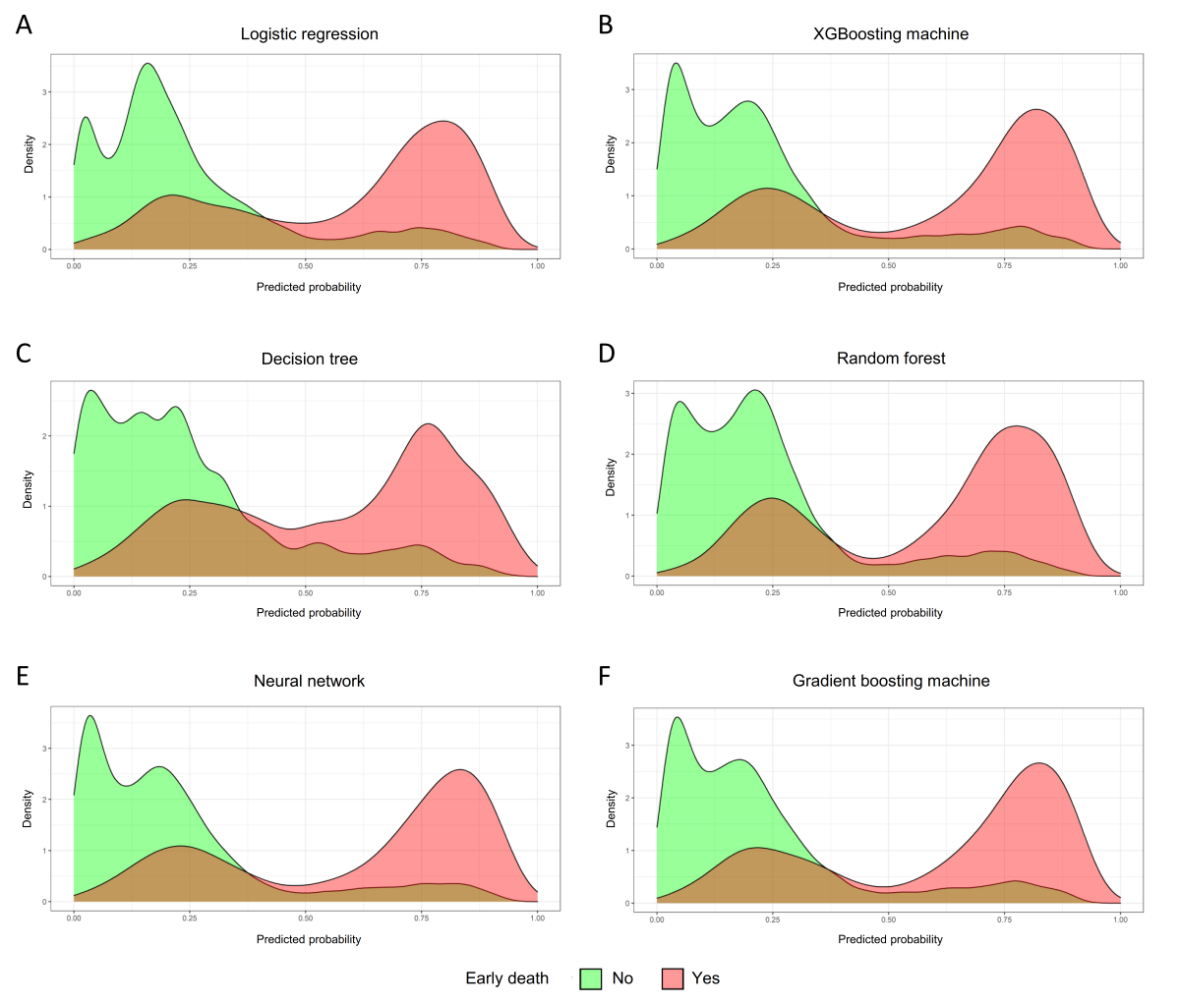
Probability curves for the different machine learning algorithms according to the presence of early death in the testing set: (A) logistic regression, (B) extreme gradient boosting (XGBoosting) machine, (C) decision tree, (D) random forest, (E) neural network, and (F) gradient boosting machine. Green curve indicates the patients without early death; red curve indicates the patients with early death.

Figure 2 shows the probability curves generated for each algorithm. Notably, the gradient boosting machine, neural network, and XGBoosting machine demonstrated significant separation between positive and negative events, with minimal overlap in the probability curves. These models exhibited the greatest distinction between patients with and without early death, indicating their superior discriminatory ability. To further quantify this discrimination, Multimedia Appendix 5 shows the discrimination slopes for each algorithm. The gradient boosting machine, neural network, and XGBoosting machine exhibited the 3 largest discrimination slopes, further supporting their superior performance in distinguishing between patients with different outcomes. Furthermore, a decision curve analysis (Multimedia Appendix 6) was conducted to evaluate the clinical usefulness of the algorithms. All the algorithms, particularly the gradient boosting machine, neural network, and XGBoosting machine, demonstrated favorable clinical utility. This analysis underscores the practical value of these models in guiding clinical decision-making.

**Figure 3:**
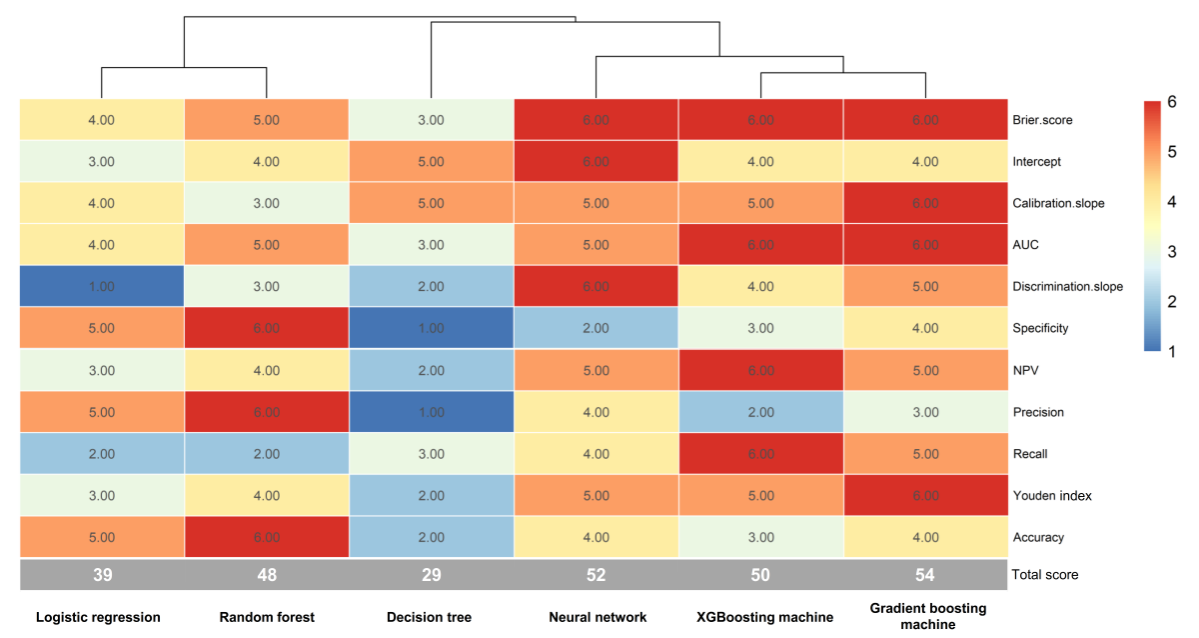
Visualization of the 11 prediction measures using a heat map in the testing set. AUC: area under curve; NPV: negative predictive value.

All evaluation metrics are summarized in Table 4. Figure 3 shows a heat map for visualizing data on prediction performance among all models, and it demonstrated that the gradient boosting machine had the highest score of prediction performance (54 points), followed by the neural network (52 points) and XGBoosting machine (50 points). These results suggest that the gradient boosting machine approach performed best in developing a prediction model to estimate the risk of early death among patients with bone metastasis.

**Table 4:** Prediction performance of machine learning approaches for predicting early death among bone metastatic patients.

Measures	Approaches					
	Logistic regression	Extreme gradient boosting machine	Decision tree	Random forest	Neural network	Gradient boosting machine
Brier score	0.147	0.142	0.157	0.143	0.142	0.142
Intercept	−0.03	−0.02	−0.01	−0.02	0.00	−0.02
Calibration slope	1.05	1.03	0.97	1.13	0.97	1.02
Area under the curve (95% CI)	0.845 (0.838-0.853)	0.858 (0.851-0.865)	0.830 (0.823-0.838)	0.856 (0.849-0.863)	0.856 (0.849-0.863)	0.858 (0.851-0.865)
Discrimination slope	0.352	0.383	0.326	0.358	0.391	0.384
Specificity	0.853	0.828	0.794	0.857	0.835	0.834
Negative predictive value	0.837	0.849	0.832	0.838	0.847	0.847
Precision	0.733	0.711	0.666	0.739	0.718	0.717
Recall	0.710	0.742	0.719	0.710	0.735	0.737
Youden index	1.562	1.570	1.513	1.567	1.570	1.571
Accuracy	0.801	0.797	0.767	0.804	0.799	0.799
Threshold	0.415	0.339	0.372	0.386	0.350	0.361
Performance score	39	50	29	48	52	54

The baseline clinical characteristics of the patients in the external validation set are summarized in Table S4 in Multimedia Appendix 1. External validation was performed using the optimal machine learning model, and the AUC was 0.847 (95% CI 0.798-0.895; Multimedia Appendix 7). Although the blue line of the calibration curve in the external validation set (Multimedia Appendix 8) deviated slightly upward from the diagonal around 0.30 in the predicted probability, it still remained close to the diagonal and showed a tendency to regress toward it. In terms of quantitative assessment, the calibration slope was 1.06 and the intercept-in-large value was 0.17, which suggests favorable calibration in the external validation.

### Model Explainability and Feature Importance

**Figure 4:**
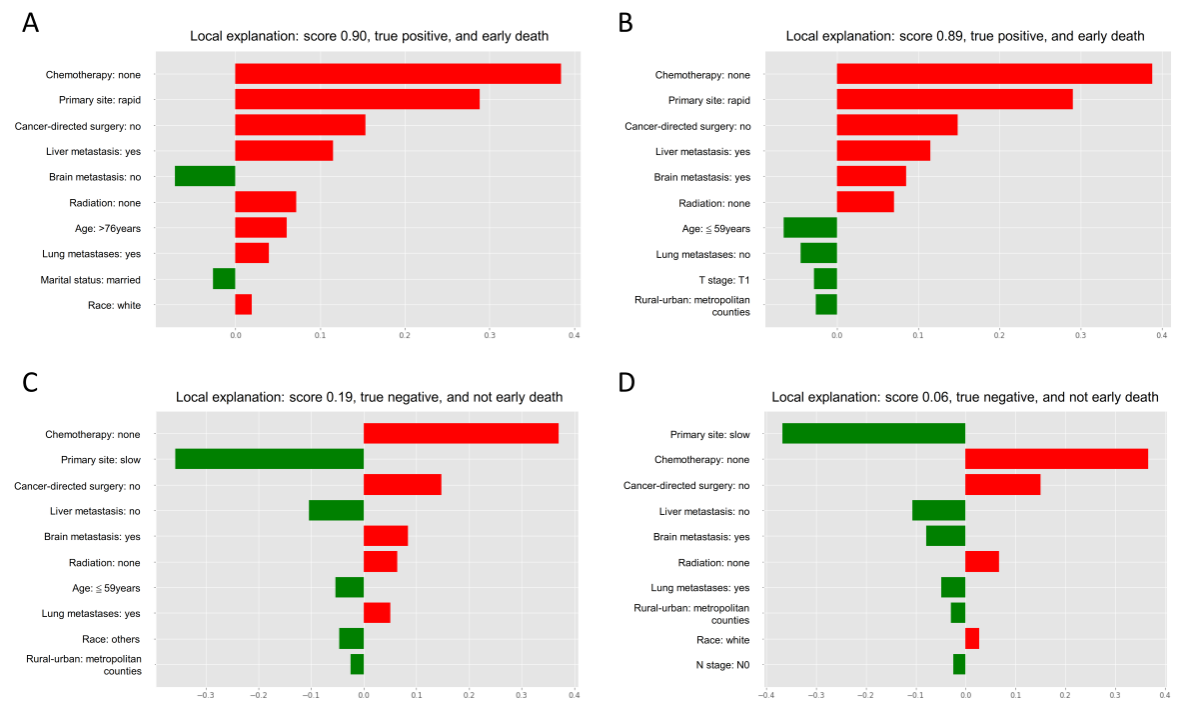
Model explanations for 4 individual cases using the Local Interpretable Model-Agnostic Explanations (LIME) technique in the testing set: (A) patients with early death, a true positive case; (B) patients with early death, a true positive case; (C) patients without early death, a true negative case; and (D) patients without early death, a true negative case. The green bars indicate protective prognostic factors, whereas the red bars represent positive contributing factors. The x-axis of the visualization illustrates the magnitude of each predictor’s impact on the final probability for the specific patient. In addition, the probability of early death for each patient is displayed in the title of each panel, providing a clear indication of the predicted outcome. N stage: node stage; T stage, tumor stage.

Model explainability was conducted to rank the variables and visualize their contributions to early death based on the gradient boosting machine algorithm. Figure 4 shows 4 individual cases in the internal validation set using LIME. Multimedia Appendix 9 shows the machine learning curve of the H_2_O method, and it depicted that error metric dependence on learning progress. The SHAP summary plot revealed that chemotherapy, primary site, and liver metastasis were the top 3 important variables associated with early death (Multimedia Appendix 10). Multimedia Appendix 11 also demonstrates the variable importance heat map, which depicted variable importance across multiple models. It demonstrated that chemotherapy, primary site, age, and liver metastasis were significantly important variables. Multimedia Appendix 12 investigates the relationship between early death and age, and it showed that early death gradually increased with age.

### Model Risk Stratification

This study used an average threshold of 37% derived from all 6 machine learning algorithms as the optimal threshold for risk stratification. On the basis of this threshold, patients were categorized into 2 distinct groups within each machine learning approach: a low-risk group and a high-risk group (Table 5). In the low-risk group, patients were identified as having a predicted risk probability ≤37%. In contrast, patients in the high-risk group had a predicted risk probability exceeding 37%. The actual probability of early death was compared between the 2 groups for each algorithm (all *P*<.001). On the basis of the risk stratification, Kaplan-Meier survival curves were plotted for each machine learning algorithm (Multimedia Appendix 13; all *P*<.001).

**Table 5:** Risk stratification of models based on machine learning among bone metastasis patients.

Approaches and groups			Probability of early death					*P* value^a^
			Predicted, %		Actual, % (4264/11,735)			
**Logistic regression**								<.001
	Low risk (≤37%)	16.74		15.5 (1123/7245)				
	High risk (>37%)	68.9		70 (3141/4490)				
**XGBoosting^b^ machine**								<.001
	Low risk (≤37%)	16.25		15.76 (1183/7504)				
	High risk (>37%)	72.85		72.82 (3081/4231)				
**Decision tree**								<.001
	Low risk (≤37%)	16.8		16.79 (1194/7111)				
	High risk (>37%)	66.91		66.39 (3070/4624)				
**Random forest**								<.001
	Low risk (≤37%)	17.6		16 (1204/7523)				
	High risk (>37%)	70.61		72.65 (3060/4212)				
**Neural network**								<.001
	Low risk (≤37%)	15.5		15.83 (1189/7510)				
	High risk (>37%)	73.39		72.78 (3075/4225)				
**Gradient boosting machine**								<.001
	Low risk (≤37%)	15.93		15.62 (1159/7420)				
	High risk (>37%)	72.32		71.96 (3105/4315)				

^a^*P* values were obtained from chi-square test.

^b^XGBoosting: extreme gradient boosting.

### Web-Based Calculator

As the gradient boosting machine was the optimal model in this study, this study further deployed an web-based calculator to predict early death among patients with bone metastasis using this model (Multimedia Appendix 14). This web-based application is available [23]. The online application could enhance the accessibility and usability of the prediction model, and it allowed health care professionals to easily input patient data and obtain valuable predictions for early mortality risk among patients with bone metastases.

## Discussion

### Principal Findings

This study developed an accurate model to predict the risk of early death among patients with bone metastasis. On systematically evaluating the prediction performance of all algorithms in this study, the model developed by the gradient boosting machine scored the highest and performed the best, followed by the neural network and XGBoosting machine in predicting early death among patients with bone metastases. Furthermore, the web-based calculator developed in this study offers a valuable tool for clinicians to make more informed clinical decisions. This information can guide treatment planning, allowing clinicians to tailor interventions based on individual patient risk profiles.

Previous studies have proposed several scoring systems to predict the survival prognosis of patients with bone metastases. For instance, Katagiri et al [8] updated a scoring system for patients with skeletal metastases by introducing a new factor and analyzing 808 patients with symptomatic skeletal metastases. Among these patients, 749 were treated nonsurgically and 59 underwent surgery for skeletal metastasis. However, the accuracy and AUC value of the model were not presented in this scoring system. Similarly, Sawada et al [24] evaluated factors associated with 30-day mortality after surgery for spinal metastasis and developed a risk scoring system based on the analysis of 3524 patients. The clinical predictors of the scoring system included sex, emergency admission, rapid-growth tumors, and nonskeletal metastasis. Other scoring systems have been designed specifically for patients with either spinal metastasis [3] or specific primary cancer [25-27]. However, these scoring systems may be subject to patient selection bias, making them less applicable to the general population of patients with bone metastases or those diagnosed with other primary cancer types. Although scoring systems tailored to specific cancers may offer greater precision, clinicians may prefer a simple and general system that can be applied across different primary sites to avoid the inconvenience associated with multiple systems [8]. In addition, some scoring systems have been exclusively designed for patients treated with surgery [3,27] or radiotherapy [28] alone. Moreover, scoring systems developed for various primary cancer types tended to focus mainly on the common primary cancers, such as lung, breast, and prostate cancer, which account for approximately 50% of all primary cancer types [29]. Consequently, other malignancies are often categorized as “others” or receive little attention in these scoring systems. Notably, these studies relied on relatively small sample sizes, ranging from tens to hundreds of patients [2,8]. Hence, the accuracy and reliability of these scoring systems may be limited owing to the restricted size of the data samples.

In light of these limitations, the development of a more accurate and comprehensive machine learning–based prediction model, as demonstrated in this study, holds significant clinical value. By using a large cohort and machine learning algorithms, this model provides a more robust and accurate assessment of the likelihood of early death among patients with bone metastasis. It offers a standardized approach that can be applied across various primary cancer types, enhancing clinical utility and facilitating personalized treatment decision-making for a broader patient population. In the entire cohort, 36.63% (43,305/118,227) of patients experienced early death. Other studies reported that the 3-month mortality rate was approximately 30% [30-32], which was consistent with this study. According to variables’ importance, this study found that chemotherapy, primary site, and liver metastasis were the top 3 important variables associated with early death. Primary tumor type and visceral metastasis have already been widely proven to be prognostic factors associated with survival outcomes in patients with bone metastasis [33]. Chemotherapy is also a significant variable related to survival [33]. Although previous studies have reported inconsistent findings regarding the association between age and survival prognosis in patients with bone metastasis [8], this study observed a gradual increase in the risk of early death with advancing age. These contrasting results could potentially be attributed to differences in age classification and the sample sizes among the various studies. In light of the findings of this study, which suggested an association between age and early death, it is important to consider age as a potential prognostic factor in clinical decision-making among patients with bone metastases.

Regarding modeling methodology, this study used 6 machine learning algorithms including logistic regression, XGBoosting machine, decision tree, random forest, neural network, and gradient boosting machine to train and optimize models [34]. These sophisticated machine learning algorithms have the capability to leverage large amounts of data and provide improved performance compared with traditional logistic regression models [13]. Machine learning approaches offer flexibility and scalability, making them suitable for various tasks such as risk stratification and survival estimation, particularly when analyzing big data [35]. Previously, machine learning has been used to develop prediction models to predict the mortality among bone metastases from specific cancer types, such as hepatocellular carcinoma [15], lung cancer [16], cancer of unknown primary site [36], and breast cancer [37]. However, our study was specifically designed to develop a machine learning model to predict the risk of early death among patients with general bone metastases. Finally, model explainability was achieved based on the gradient boosting machine algorithm in this study to improve model transparency and user trust because it could not only show the risk probability of early death among individual patients but also provide reasonable explanations behind it [38]. The model aimed to assess the probability of early death among patients with bone metastasis, and it could be used at different time points depending on the clinical scenario and the patient’s treatment history. For instance, it can be used to assess the probability of early death in patients newly diagnosed with bone metastasis, aiding in treatment decision-making. Similarly, for patients who have undergone medical oncology therapies or are considering surgery, the model can offer additional information regarding the likelihood of early death, thus assisting in the selection of the most appropriate treatment approach.

### Limitations

This study had some limitations. First, the number of some primary cancers, such as penis and eye and orbit, was very small, possibly owing to the low incidence rate of bone metastasis among those cancers. Thus, it might be difficult to draw a conclusion to guide clinical decision among patients with those primary cancers. Second, only a small fraction of patients (11,532/118,227, 9.75%) underwent cancer-directed surgery, possibly owing to the increasing use of antiosteolytic drugs, such as bisphosphonates and denosumab, which largely reduced skeletal-related events. Thus, validation of the model among a sufficient number of surgically treated patients was necessary. Third, some laboratory factors [39] such as albumin, hemoglobin, and lymphocytes or the Eastern Cooperative Oncology Group score [40] were not considered for analysis in the study because of the unavailability of these data in the SEER database. Therefore, although our model obtained favorable prediction in the internal and external validation, clinical decision should not rely on survival estimation alone; it also needs a complete evaluation of the patient’s pain, neurological impairment, and general performance status.

### Conclusions

This study developed a machine learning–based prediction model to accurately assess the probability of early death among patients with bone metastasis. The gradient boosting machine demonstrated the highest prediction performance among the 6 approaches evaluated in this study. Age, sex, race, marital status, rural-urban continuum, primary site, T stage, N stage, brain metastasis, liver metastasis, lung metastasis, cancer-directed surgery, radiation, and chemotherapy were identified as significant factors associated with early death. The model’s explainability, using LIME, provides insights into the contributions of these variables to early death. External validation of the model demonstrated its robustness and reliability. This prediction model, which is presented in the format of an online application, has the potential to guide clinical decision-making and improve the care of patients with bone metastasis by identifying those at a higher risk of early death. However, further research and clinical judgment are necessary to determine the appropriate treatment options for individual patients.

## Appendix

Multimedia Appendix 1 Classification of primary cancer, introduction to the 6 machine learning algorithms, hyperparameters, and patients' baseline characteristics in the external validation set.

Multimedia Appendix 2 The incidence of early death over the period of inclusion.

Multimedia Appendix 3 Receiver operating curves for the 6 approaches in the testing set: (A) logistic regression, (B) extreme gradient boosting machine, (C) decision tree, (D) random forest, (E) neural network, and (F) gradient boosting machine.

Multimedia Appendix 4 Calibration curves for the 6 approaches in the testing set: (A) logistic regression, (B) extreme gradient boosting machine, (C) decision tree, (D) random forest, (E) neural network, and (F) gradient boosting machine.

Multimedia Appendix 5 Box plots of the predicted probabilities of early death between patients with (yes) and without (no) actual early death in the testing set: (A) logistic regression, (B) extreme gradient boosting machine, (C) decision tree, (D) random forest, (E) neural network, and (F) gradient boosting machine.

Multimedia Appendix 6 Decision curve analysis for the 6 approaches in the testing set: (A) logistic regression, (B) extreme gradient boosting machine, (C) decision tree, (D) random forest, (E) neural network, and (F) gradient boosting machine. In the analysis, a horizontal gray line is used to represent the “treated-for-none” scheme, indicating the approach where no treatment is administered. In addition, another reference line is included to represent the “treated-for-all” scheme. This line represents the approach where all patients receive treatment regardless of their risk or prognosis.

Multimedia Appendix 7 Receiver operating curve for the gradient boosting machine in the external validation set.

Multimedia Appendix 8 Calibration curve for the gradient boosting machine in the external validation set.

Multimedia Appendix 9 Learning curve for automatic machine learning. The plot is able to identify whether the model was overfitting or underfitting. The training and validation curves converged in the analysis, indicating favorable fitting.

Multimedia Appendix 10 Evaluation of feature importance using SHAP (Shapley Additive Explanations): (A) the training group and (B) the testing group. In the SHAP summary plot, the y-axis is the features, and the x-axis is the Shapley value in each case. Color represents the eigenvalue (red indicates high and blue indicates low), and color enables us to match how the change of eigenvalue affects the change of risk. The overlapping points jitter in the direction of y-axis; it could enable us to understand how the Shapley values distribute in each feature, and these features were sorted according to their importance.

Multimedia Appendix 11 Variable importance heat map based on a series of gradient boosting machine learning models. The models and variables are ordered by their similarity.

Multimedia Appendix 12 The association between age and response (probability of early death).

Multimedia Appendix 13 The Kaplan-Meier survival curves plotted to illustrate the risk stratification achieved by the different machine learning algorithms in the testing set: (A) logistic regression, (B) extreme gradient boosting machine, (C) decision tree, (D) random forest, (E) neural network, and (F) gradient boosting machine. In each plot, the blue line represents the low-risk group, whereas the red line represents the high-risk group. The significant separation between the 2 groups, as indicated by the log-rank test (*P*<.001), highlights the ability of the machine learning algorithms to effectively stratify patients based on their risk of early death.

Multimedia Appendix 14 The web-based calculator. The calculator includes a panel for users to select model parameters, a section for showing predicted early mortality, a section for introducing model information, and a section for recommending therapeutic strategies.

## Abbreviations

AUC: area under the curve
LIME: Local Interpretable Model-Agnostic Explanations
N stage: node stage
NX: unknown node stage
SEER: Surveillance, Epidemiology, and End Results
SHAP: Shapley Additive Explanations
T stage: tumor stage
TX: unknown tumor stage
XGBoosting: extreme gradient boosting

## Data Availability

On reasonable request, data can be obtained from the corresponding author. The Surveillance, Epidemiology, and End Results (SEER) data are available [41]. We obtained approval to access the SEER of the National Cancer Institute in the United States using the reference number 23489-Nov2020.
