# Supplementary material for: A Web-Based Calculator to Predict Early Death Among Patients With Bone Metastasis Using Machine Learning Techniques: Development and Validation Study
Source: J Med Internet Res. 2023 Oct 23;25:e47590. doi: 10.2196/47590 (PMC10628690; doi:10.2196/47590)
Supplement: Multimedia Appendix 1 [file jmir_v25i1e47590_app1.docx]

| **Table S1**. Classification of primary cancer and corresponding number of patients in the whole bone metastasis cohort. | | |
| --- | --- | --- |
| Primary site | Number | Type |
| Anus, Anal Canal and Anorectum | 107 | R |
| Appendix | 36 | R |
| Ascending Colon | 274 | R |
| Bones and Joints | 167 | M |
| Breast |  |  |
| Positive ER status | 12184 | S |
| Others | 3825 | M |
| Cecum | 372 | R |
| Cervix Uteri | 558 | R |
| Corpus Uteri | 696 | M |
| Descending Colon | 89 | R |
| Esophagus | 2072 | R |
| Eye and Orbit | 20 | R |
| Floor of Mouth | 33 | R |
| Gallbladder | 189 | R |
| Gum and Other Mouth | 87 | R |
| Hepatic Flexure | 76 | R |
| Hypopharynx | 66 | R |
| Intrahepatic Bile Duct | 442 | R |
| Kidney and Renal Pelvis | 5279 | M |
| Large Intestine, NOS | 404 | R |
| Larynx | 105 | R |
| Lip | 3 | R |
| Liver | 1847 | R |
| Lung and Bronchus | 56491 | R |
| Melanoma of the Skin | 1211 | R |
| Mesothelioma | 219 | M |
| Miscellaneous | 7 | M |
| Nasopharynx | 218 | R |
| NHL - Extranodal | 25 | S |
| Nose, Nasal Cavity and Middle Ear | 75 | R |
| Oropharynx | 49 | R |
| Other Biliary | 190 | R |
| Other Endocrine including Thymus | 88 | M |
| Other Female Genital Organs | 11 | M |
| Other Male Genital Organs | 4 | M |
| Other Myeloid/Monocytic Leukemia | 1 | S |
| Other Non-Epithelial Skin | 74 | M |
| Other Urinary Organs | 32 | R |
| Ovary | 400 | M |
| Pancreas | 2857 | R |
| Penis | 11 | M |
| Peritoneum, Omentum and Mesentery | 27 | R |
| Pleura | 2 | M |
| Prostate | 19489 | S |
| Rectosigmoid Junction | 255 | R |
| Rectum | 824 | R |
| Retroperitoneum | 40 | R |
| Salivary Gland | 187 | M |
| Sigmoid Colon | 526 | R |
| Small Intestine | 162 | R |
| Soft Tissue including Heart | 662 | M |
| Splenic Flexure | 31 | R |
| Stomach | 1843 | R |
| Testis | 144 | M |
| Thyroid | 611 | S |
| Tongue | 170 | R |
| Tonsil | 119 | R |
| Trachea, Mediastinum and Other Respiratory Organs | 24 | M |
| Transverse Colon | 124 | R |
| Ureter | 125 | R |
| Urinary Bladder | 1732 | R |
| Uterus, NOS | 141 | M |
| Vagina | 43 | M |
| Vulva | 53 | M |
| ER, Estrogen receptor; NOS, not otherwise specified; NHL, non-Hodgkin’s lymphoma; R, Rapid growth; M, Moderate growth; S, Slow growth. | | |

| **Table S2.** The introduction of the six machine learning algorithms used in the study. |
| --- |
| **Logistic regression** |
| Logistic regression is a widely used classification algorithm that predicts the probability of a binary outcome. It models the relationship between the dependent variable and independent variables by estimating the probabilities using a logistic function. |
| **Extreme gradient boosting machine** |
| Extreme gradient boosting machine is a powerful machine learning algorithm that is used for both regression and classification tasks. It is an optimized implementation of the gradient boosting machine algorithm, and builds an ensemble of weak prediction models (decision trees) and iteratively improves the model by minimizing the objective function using gradient descent. |
| **Decision tree** |
| Decision tree is a flowchart-like model that represents decisions and their possible consequences. It is a supervised learning algorithm that uses a tree-like structure to make predictions by splitting the data based on different features. Decision trees are easy to interpret and can handle both categorical and numerical data. |
| **Random forest** |
| Random Forest is an ensemble learning algorithm that combines multiple decision trees to make predictions. It works by creating a set of decision trees on different subsets of the training data and then combining their predictions. Random Forest improves the accuracy and generalization of the model by reducing overfitting and increasing robustness. |
| **Neural network** |
| Neural networks are a class of machine learning algorithms inspired by the human brain's structure and functioning. They consist of interconnected nodes (neurons) organized in layers. Neural networks can learn complex patterns and relationships in the data by adjusting the weights between the neurons during the training process. They are widely used for various tasks such as image recognition, natural language processing, and prediction. |
| **Gradient boosting machine** |
| Gradient Boosting Machine (GBM) is an ensemble learning algorithm that combines multiple weak learning models (usually decision trees) to create a strong predictive model. GBM builds the model in a stage-wise manner, where each new model is trained to correct the mistakes made by the previous models. It is a powerful algorithm that can handle various types of data and is known for its high predictive accuracy. |

| **Table S3**. Machine learning approaches and model hyper-parameters. | |
| --- | --- |
| **Approaches** | **Parameters** |
| Logistic regression | C=0.01, random_state=42. |
| Extreme gradient boosting machine | base_score=0.5, booster='gbtree', colsample_bylevel=1, colsample_bynode=1, colsample_bytree=1, enable_categorical=False, gamma=0, gpu_id=-1, importance_type=None, interaction_constraints='', learning_rate=0.125, max_delta_step=0, max_depth=4, min_child_weight=9, missing=nan, monotone_constraints='()', n_estimators=119, n_jobs=8, num_parallel_tree=1, predictor='auto', random_state=42, reg_alpha=0, reg_lambda=1, scale_pos_weight=1, subsample=1, tree_method='exact', use_label_encoder=False, validate_parameters=1, verbosity=None. |
| Decision tree | max_depth=42, max_features='log2', min_samples_leaf=55, min_samples_split=192, random_state=42. |
| Random forests | max_depth=51, min_samples_leaf=22, min_samples_split=74, n_estimators=39, random_state=42. |
| Neural network | random_state=42,activation='relu',alpha=0.0001,batch_size='auto',beta_1=0.9, beta_2=0.999, early_stopping=False,epsilon=1e-08,hidden_layer_sizes=(100), learning_rate='constant', learning_rate_init=0.001,max_iter=200, momentum=0.9, n_iter_no_change=10, nesterovs_momentum=True, power_t=0.5,shuffle=True, tol=0.0001, validation_fraction=0.1,verbose=False, warm_start=False. |
| Gradient boosting machines | max_depth=4, max_features='log2', min_samples_leaf=135, min_samples_split=55, n_estimators=185, random_state=42. |

| **Table S4.** Patient’s baseline characteristics in the external validation set and SEER database. | | |
| --- | --- | --- |
| Characteristics | External validation set | SEER database |
| n | 332 | 118227 |
| Age (years, mean (SD)) | 62.44 (13.79) | 67.10 (12.39) |
| Sex (%) |  |  |
| Female | 113 (34.0) | 49931 (42.2) |
| Male | 219 (66.0) | 68296 (57.8) |
| Race ^a^ (%) |  |  |
| Black | 0 (0.0) | 14135 (12.0) |
| White | 0 (0.0) | 94061 (79.6) |
| Others | 332 (100.0) | 9715 (8.2) |
| Unknown | 0 (0.0) | 316 (0.3) |
| Marital status (%) |  |  |
| Divorced | 25 (7.5) | 13413 (11.3) |
| Married (including common law) | 208 (62.7) | 60812 (51.4) |
| Separated | 3 (0.9) | 1479 (1.3) |
| Single (never married) | 48 (14.5) | 20419 (17.3) |
| Widowed | 9 (2.7) | 15946 (13.5) |
| Unmarried or Domestic Partner | 1 (0.3) | 438 (0.4) |
| Unknown | 38 (11.4) | 5720 (4.8) |
| Rural urban (%) |  |  |
| Metropolitan counties | 264 (79.5) | 101904 (86.2) |
| Nonmetropolitan counties | 56 (16.9) | 16175 (13.7) |
| Unknown | 12 (3.6) | 148 (0.1) |
| Primary site (%) |  |  |
| Slow growth | 72 (21.7) | 32309 (27.3) |
| Moderate growth | 29 (8.7) | 12037 (10.2) |
| Rapid growth | 231 (69.6) | 73881 (62.5) |
| T stage (%) |  |  |
| T0 | 5 (1.5) | 1730 (1.5) |
| T1 | 34 (10.2) | 15293 (12.9) |
| T2 | 72 (21.7) | 25004 (21.1) |
| T3 | 68 (20.5) | 20998 (17.8) |
| T4 | 101 (30.4) | 28169 (23.8) |
| TX | 52 (15.7) | 27033 (22.9) |
| N stage (%) |  |  |
| N0 | 86 (25.9) | 35017 (29.6) |
| N1 | 84 (25.3) | 24271 (20.5) |
| N2 | 99 (29.8) | 27961 (23.7) |
| N3 | 34 (10.2) | 14592 (12.3) |
| NX | 29 (8.7) | 16386 (13.9) |
| Brain metastases (%) |  |  |
| No | 263 (79.2) | 98208 (83.1) |
| Unknown | 16 (4.8) | 3955 (3.3) |
| Yes | 53 (16.0) | 16064 (13.6) |
| Liver metastases (%) |  |  |
| No | 244 (73.5) | 83629 (70.7) |
| Unknown | 9 (2.7) | 3151 (2.7) |
| Yes | 79 (23.8) | 31447 (26.6) |
| Lung metastases (%) |  |  |
| No | 217 (65.4) | 83055 (70.3) |
| Unknown | 14 (4.2) | 4272 (3.6) |
| Yes | 101 (30.4) | 30900 (26.1) |
| Cancer directed surgery (%) |  |  |
| No | 268 (80.7) | 106096 (89.7) |
| Unknown | 48 (14.5) | 599 (0.5) |
| Yes | 16 (4.8) | 11532 (9.8) |
| Radiation (%) |  |  |
| None/Unknown | 200 (60.2) | 71422 (60.4) |
| Yes | 132 (39.8) | 46805 (39.6) |
| Chemotherapy (%) |  |  |
| None/Unknown | 154 (46.4) | 63740 (53.9) |
| Yes | 178 (53.6) | 54487 (46.1) |
| SD, Standard deviation; T stage, Tumor stage; N stage, node stage.  ^a^ All patients were classified as "Others" in terms of race, as they were all of yellow race. | | |
