# Supplementary figures and images for: A Web-Based Calculator to Predict Early Death Among Patients With Bone Metastasis Using Machine Learning Techniques: Development and Validation Study
Source: J Med Internet Res. 2023 Oct 23;25:e47590. doi: 10.2196/47590 (PMC10628690; doi:10.2196/47590)

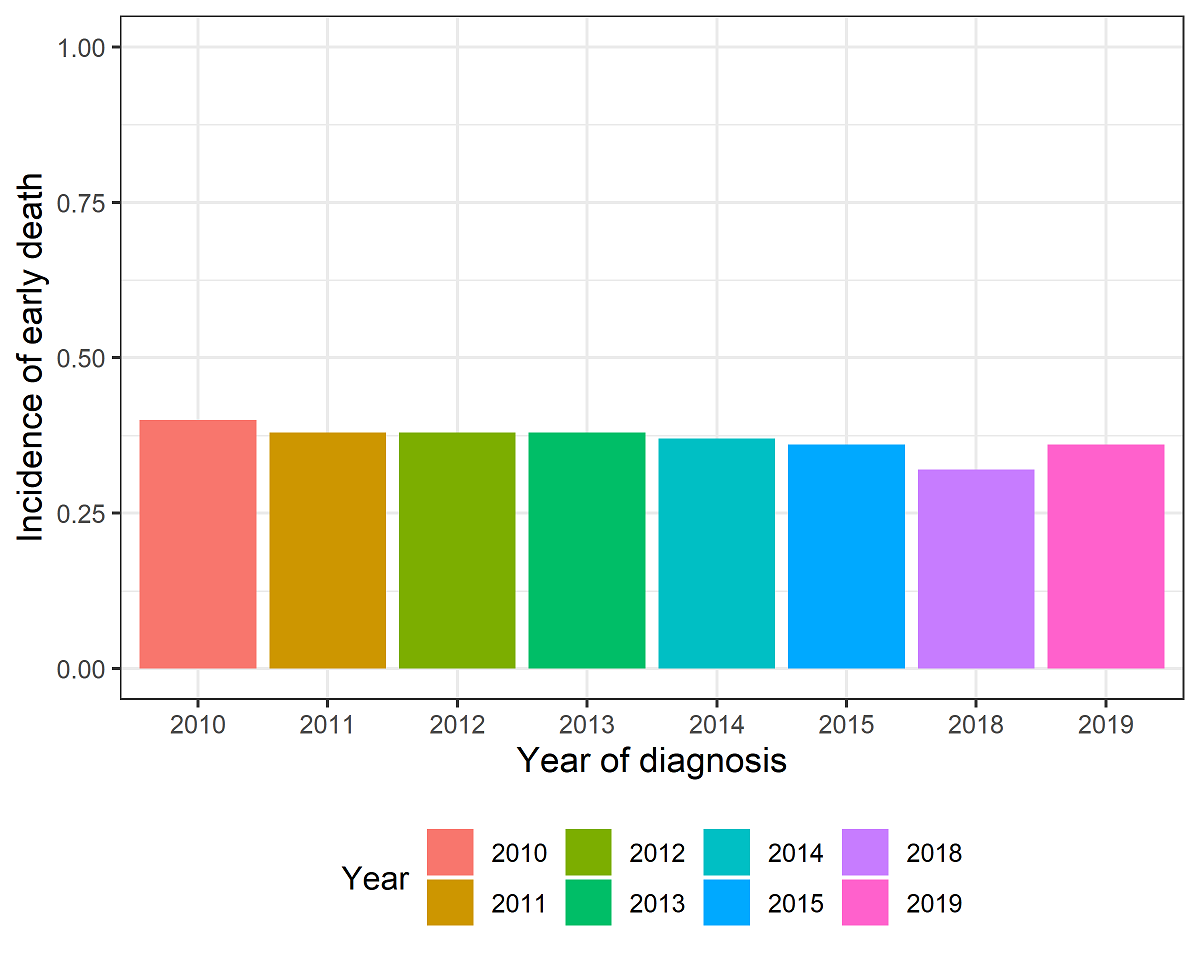

Supplement: Multimedia Appendix 2 [file jmir_v25i1e47590_app2.png]

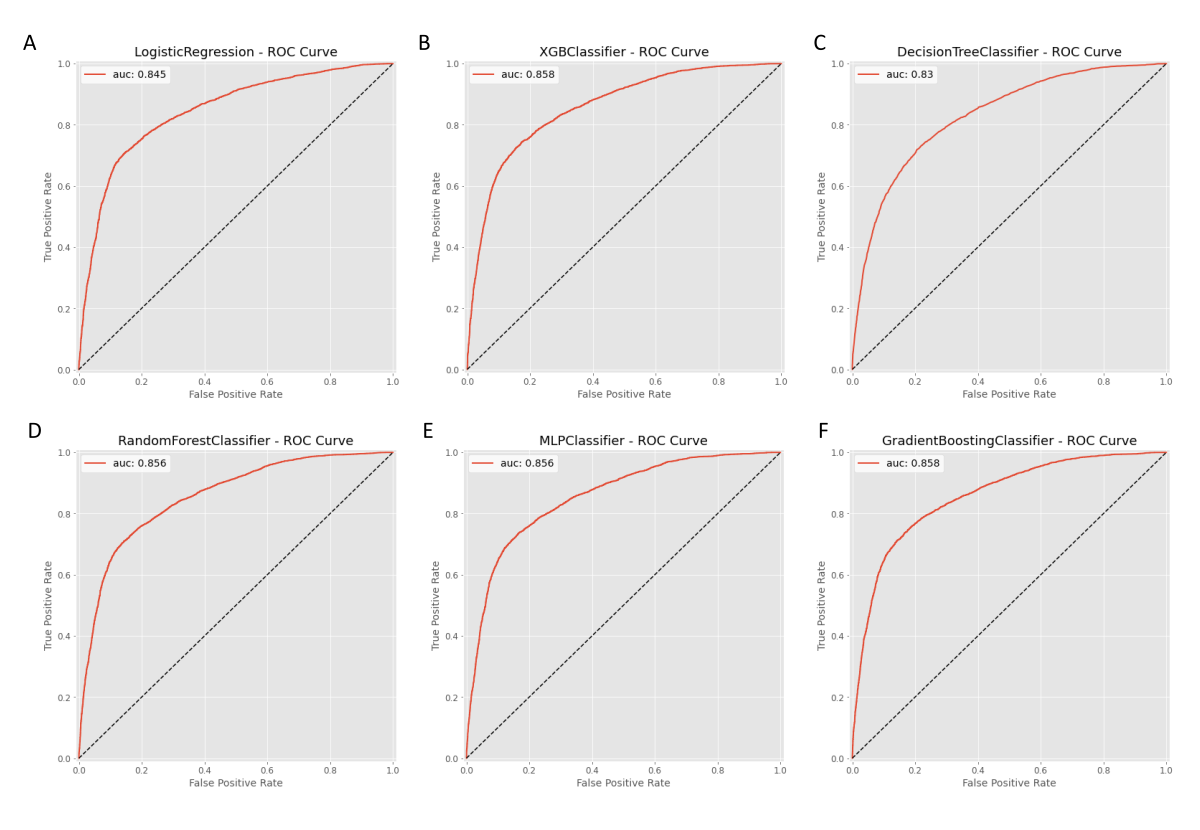

Supplement: Multimedia Appendix 3 [file jmir_v25i1e47590_app3.png]

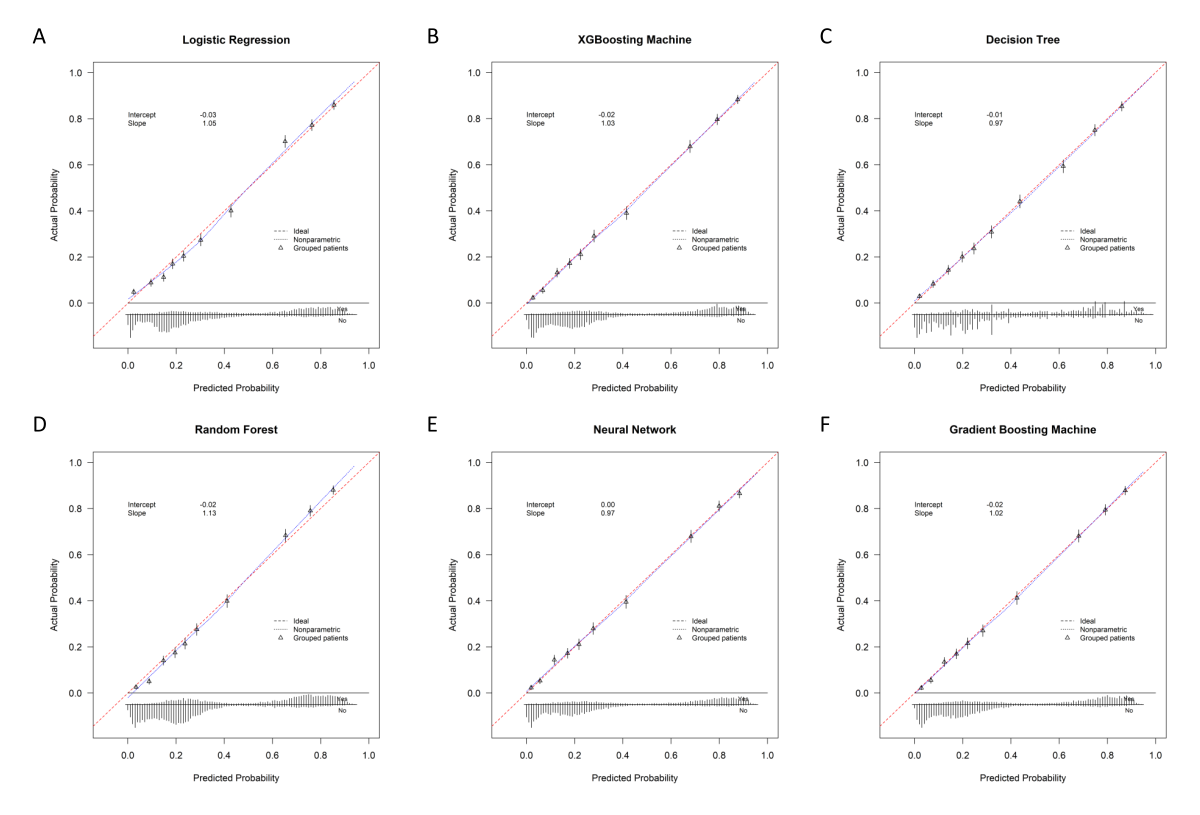

Supplement: Multimedia Appendix 4 [file jmir_v25i1e47590_app4.png]

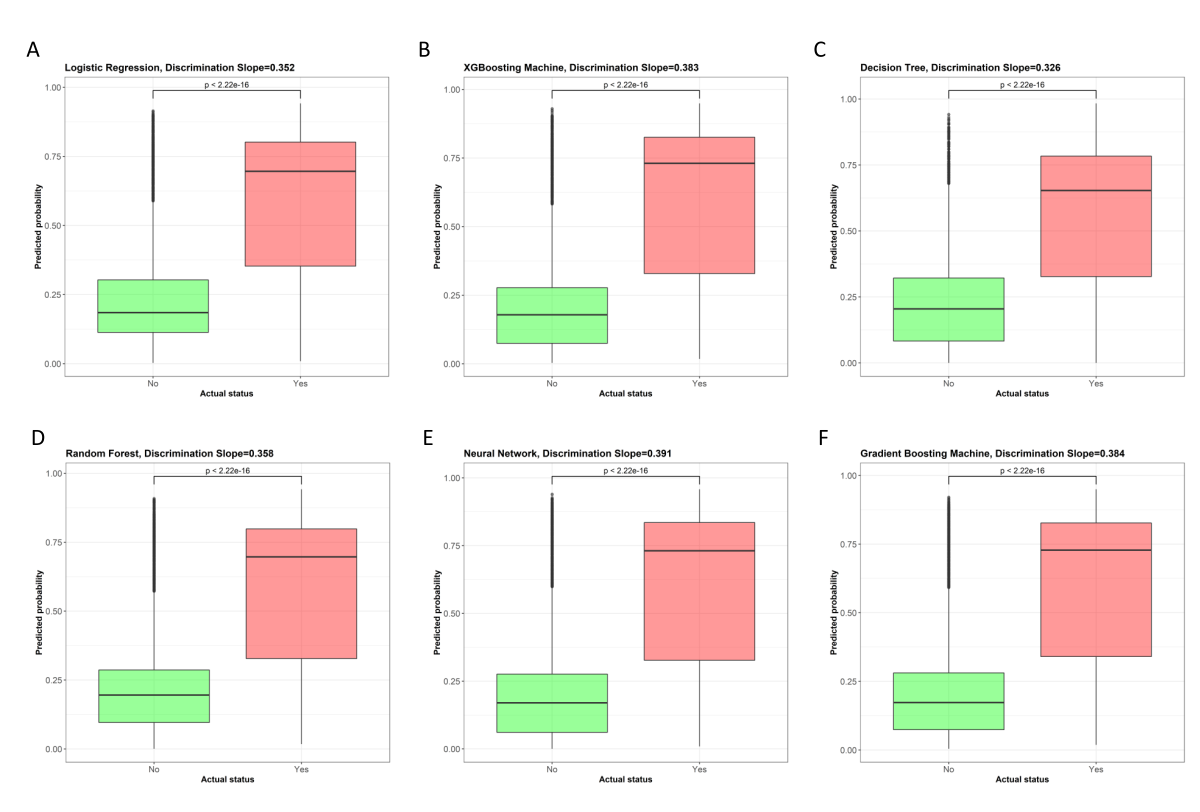

Supplement: Multimedia Appendix 5 [file jmir_v25i1e47590_app5.png]

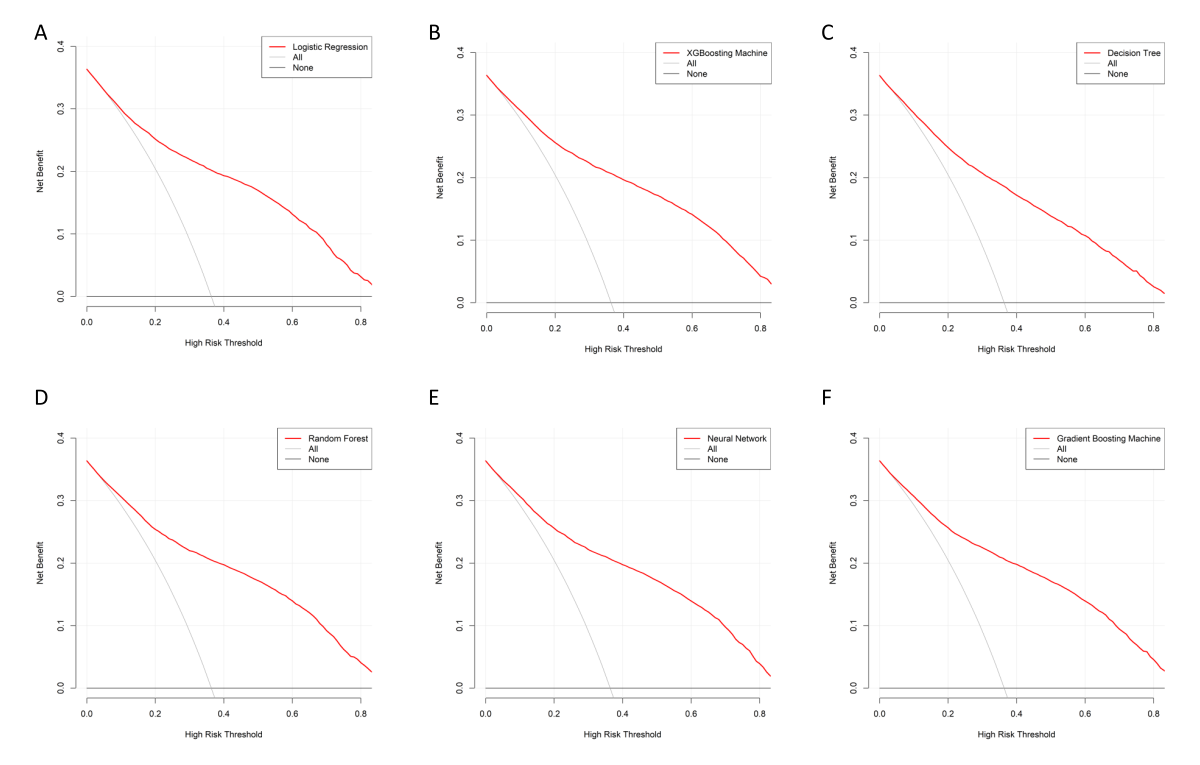

Supplement: Multimedia Appendix 6 [file jmir_v25i1e47590_app6.png]

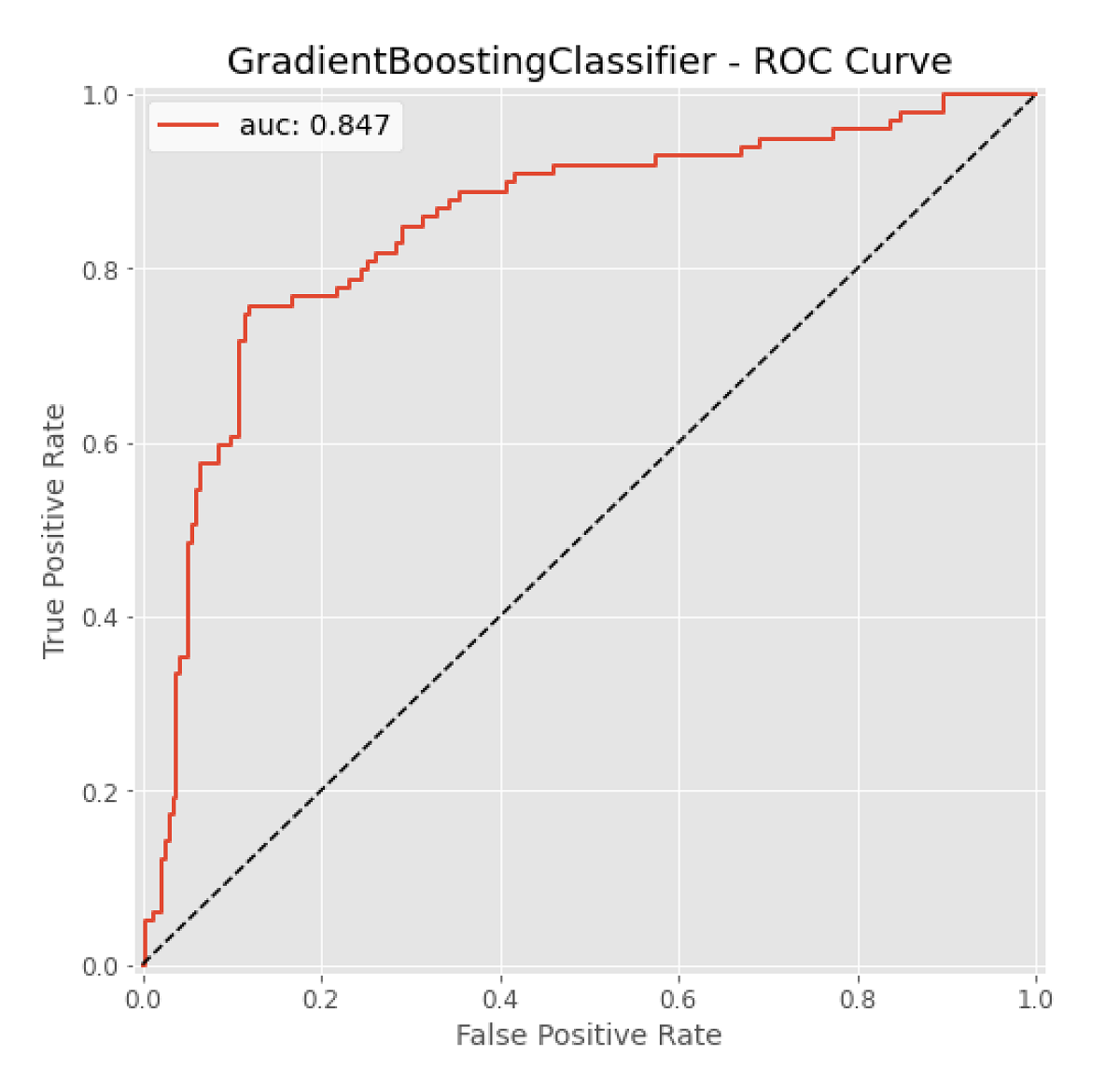

Supplement: Multimedia Appendix 7 [file jmir_v25i1e47590_app7.png]

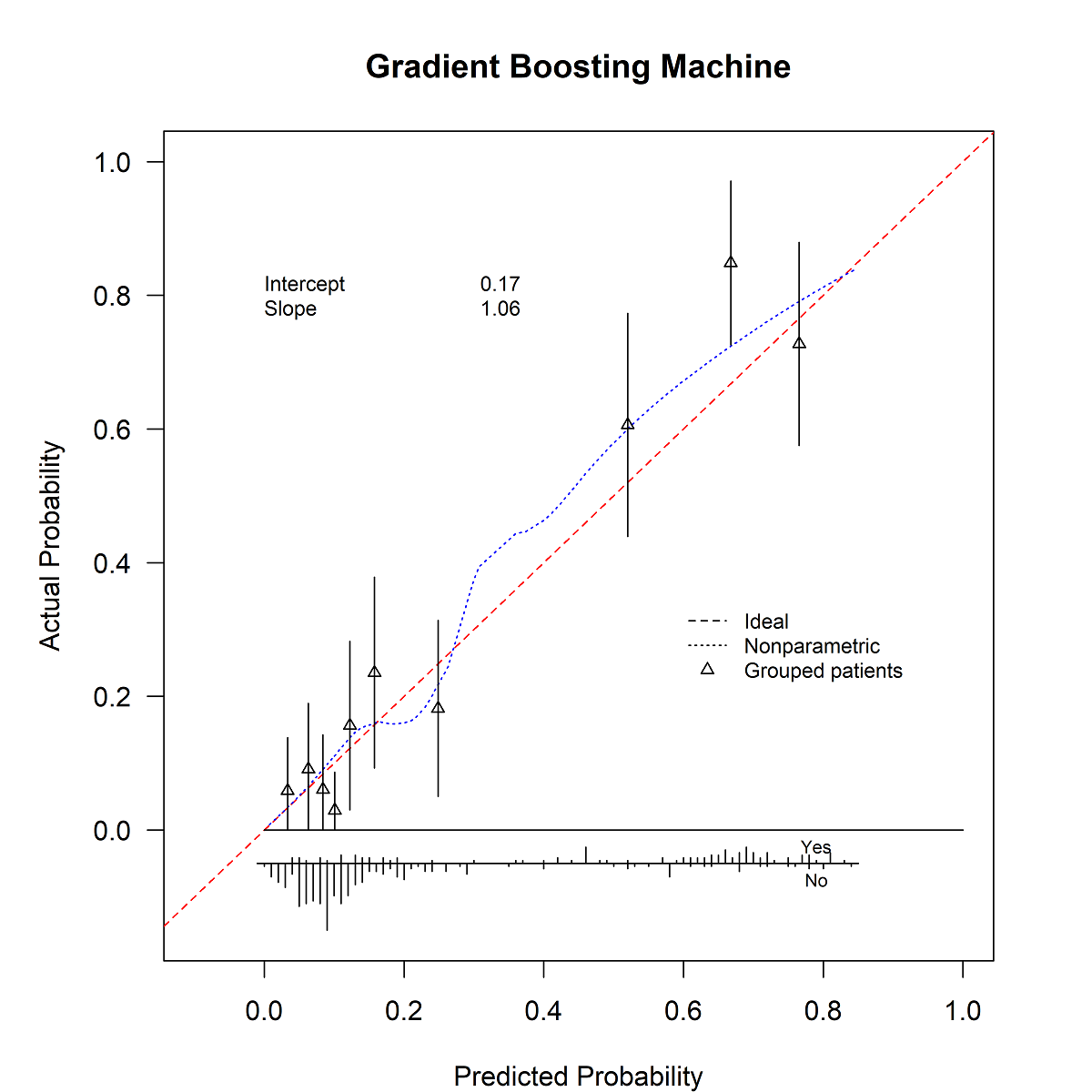

Supplement: Multimedia Appendix 8 [file jmir_v25i1e47590_app8.png]

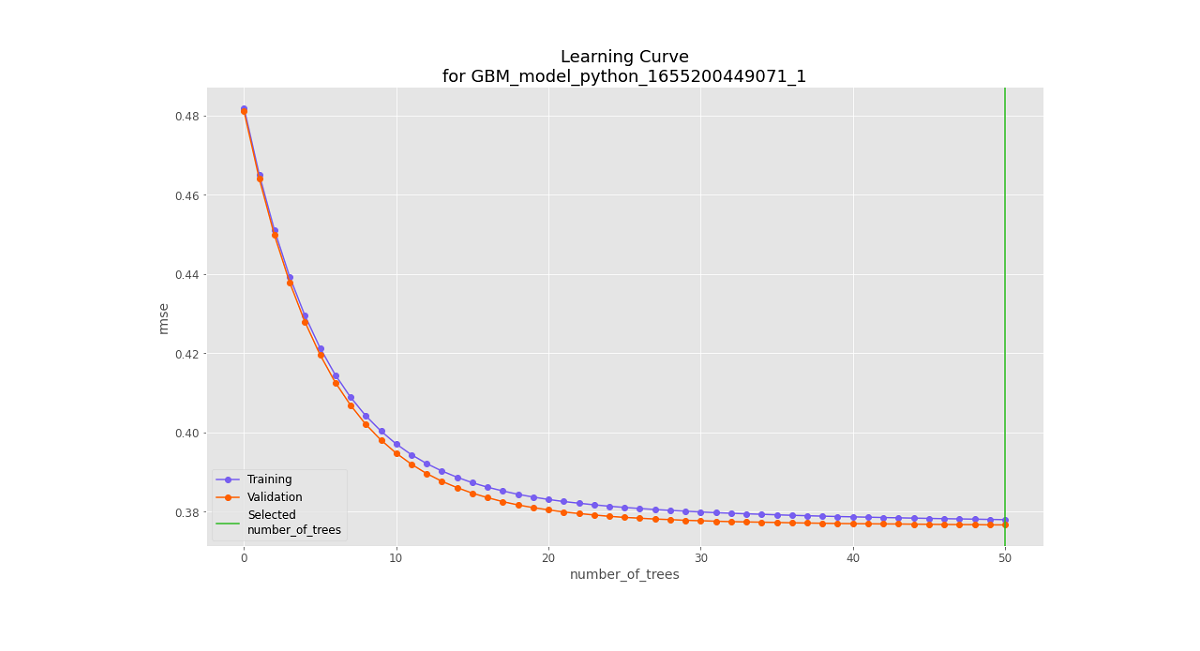

Supplement: Multimedia Appendix 9 [file jmir_v25i1e47590_app9.png]

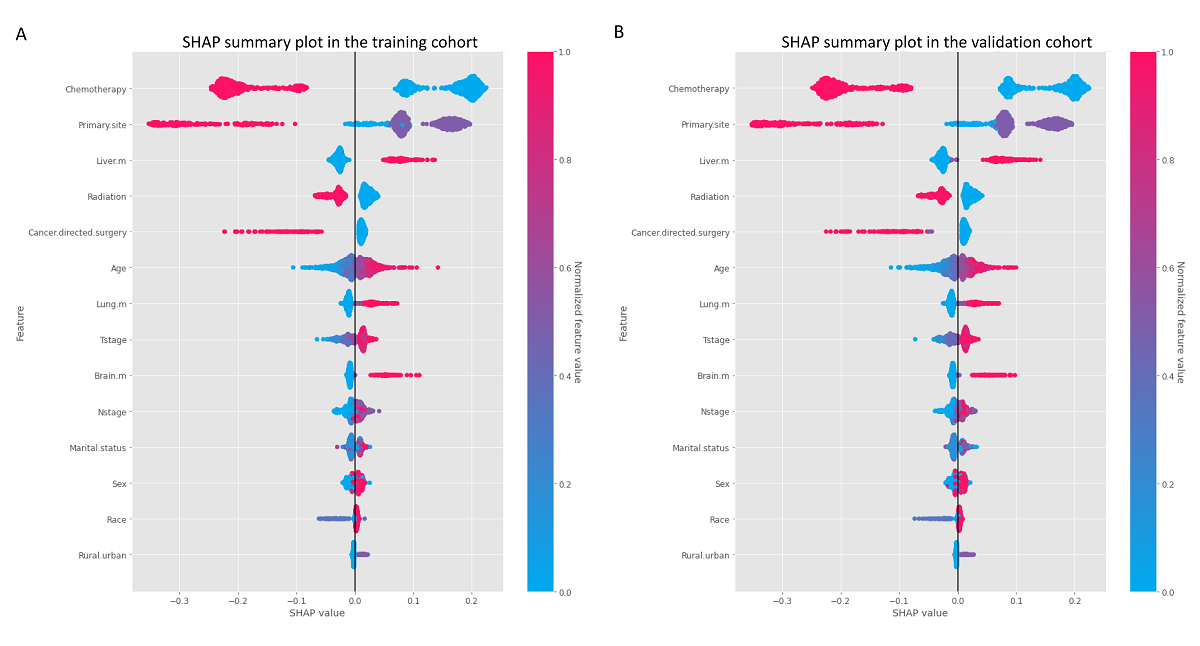

Supplement: Multimedia Appendix 10 [file jmir_v25i1e47590_app10.png]

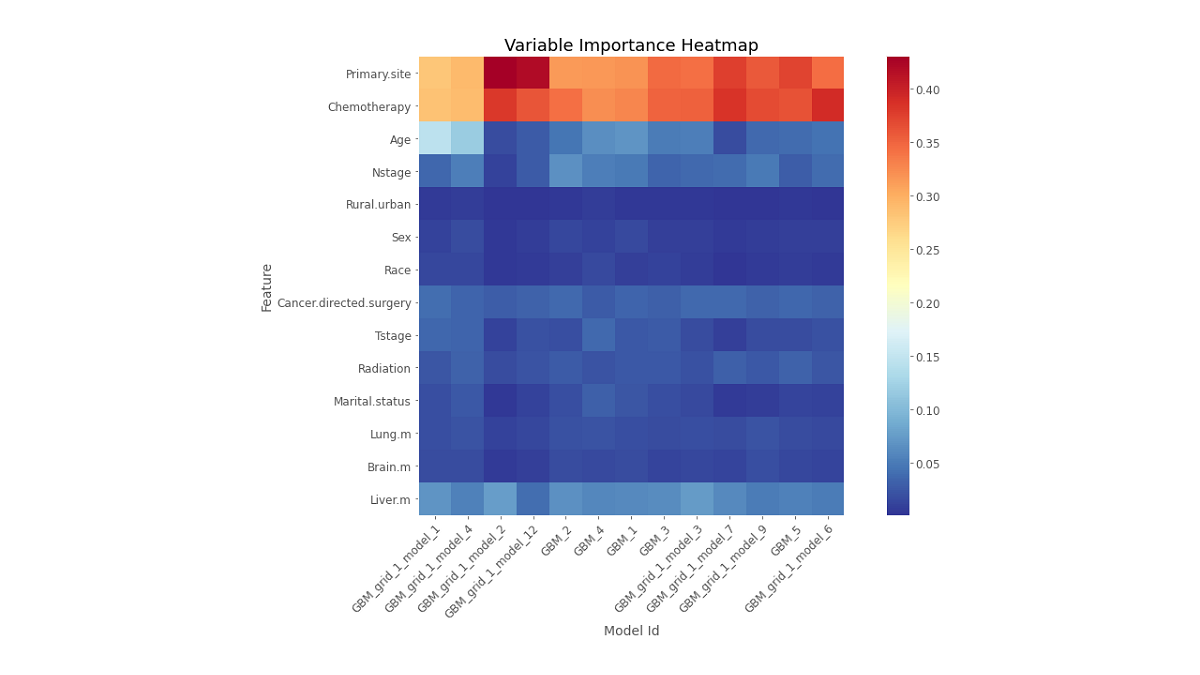

Supplement: Multimedia Appendix 11 [file jmir_v25i1e47590_app11.png]

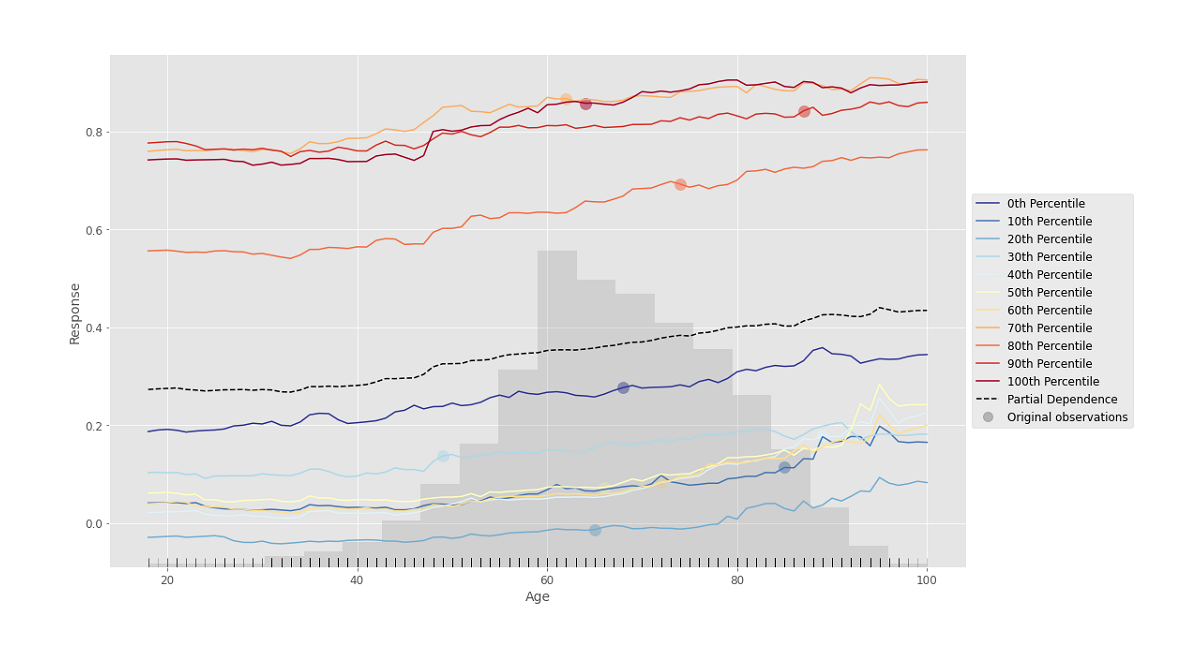

Supplement: Multimedia Appendix 12 [file jmir_v25i1e47590_app12.png]

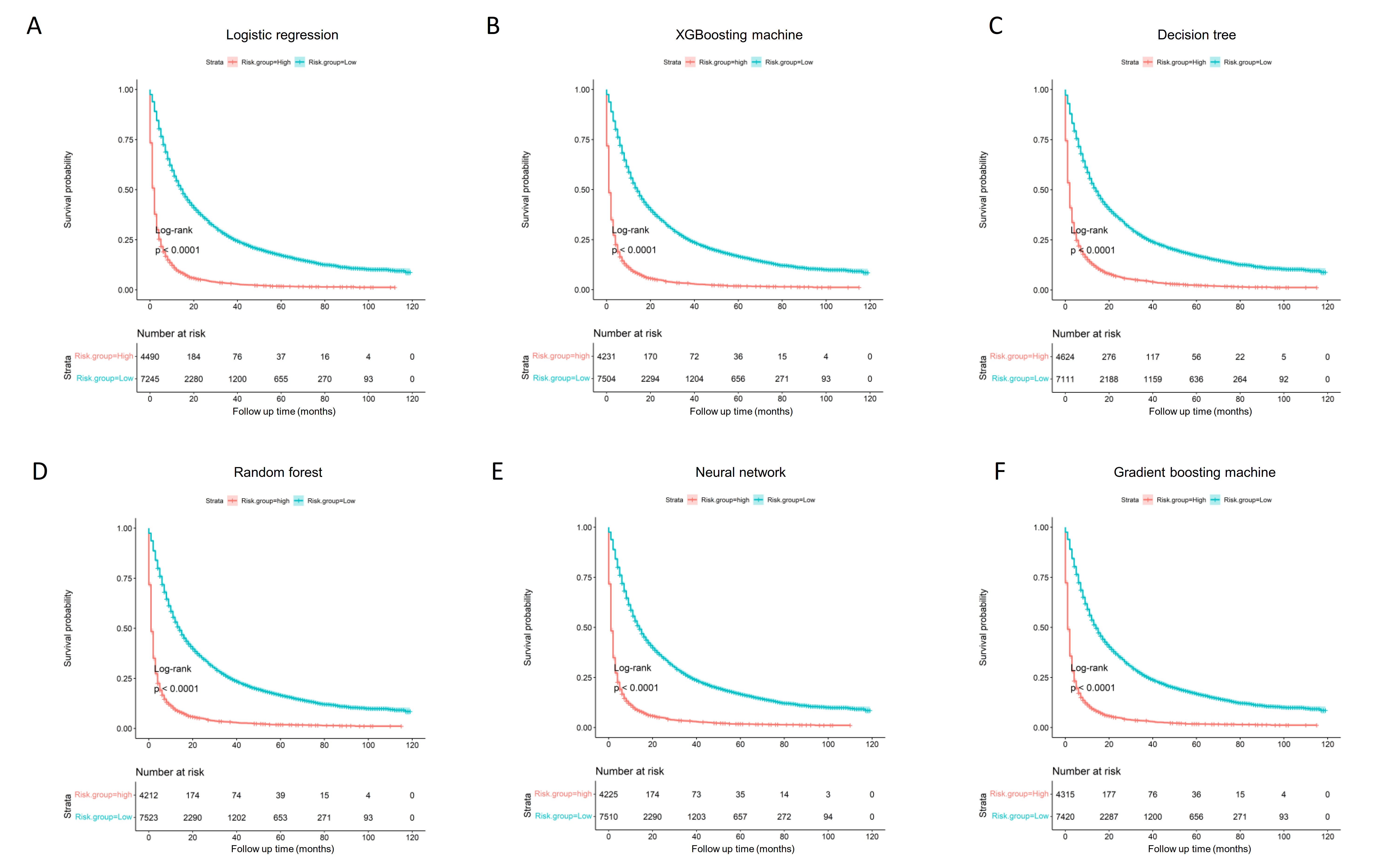

Supplement: Multimedia Appendix 13 [file jmir_v25i1e47590_app13.png]

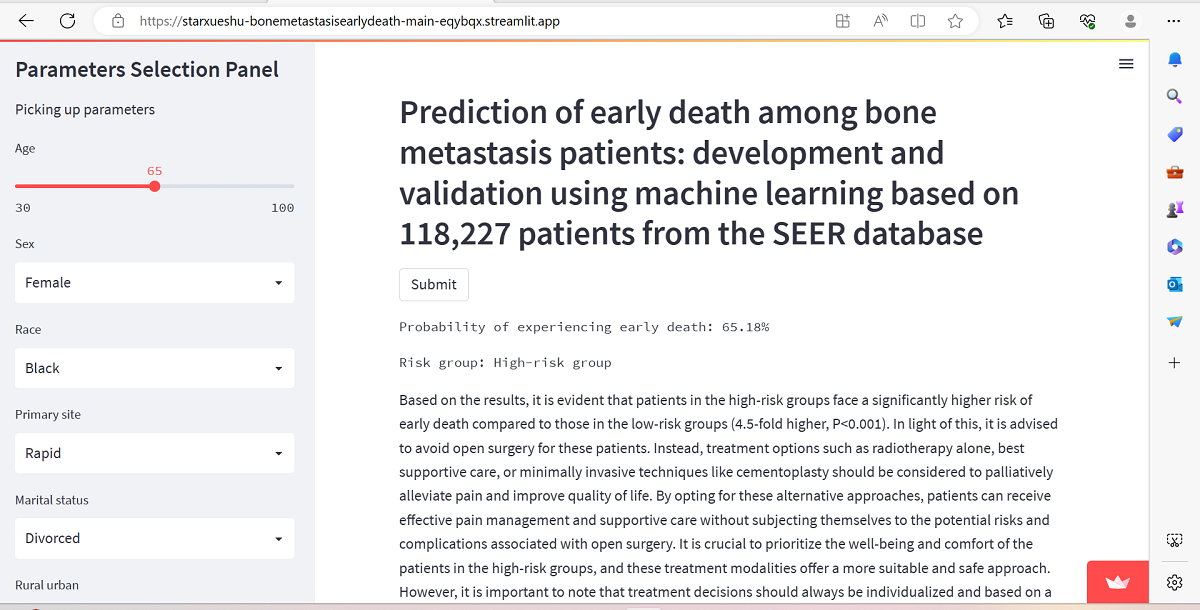

Supplement: Multimedia Appendix 14 [file jmir_v25i1e47590_app14.png]
